# Dystrophin Is Required for the Proper Timing in Retinal Histogenesis: A Thorough Investigation on the *mdx* Mouse Model of Duchenne Muscular Dystrophy

**DOI:** 10.3389/fnins.2020.00760

**Published:** 2020-08-31

**Authors:** Irene Persiconi, Francesca Cosmi, Noemi Antonella Guadagno, Giuseppe Lupo, Maria Egle De Stefano

**Affiliations:** ^1^Department of Biology and Biotechnology “Charles Darwin”, Sapienza University of Rome, Rome, Italy; ^2^Department of Biosciences, University of Oslo, Oslo, Norway; ^3^Laboratory Affiliated to Istituto Pasteur Italia-Fondazione Cenci Bolognetti, Sapienza University of Rome, Rome, Italy; ^4^Center for Research in Neurobiology “Daniel Bovet”, Sapienza University of Rome, Rome, Italy

**Keywords:** Duchenne muscular dystrophy, *mdx* mice, dystrophin, retinogenesis, retinal ganglion cells, GABA, amacrine cells

## Abstract

Duchenne muscular dystrophy (DMD) is a lethal X-linked muscular disease caused by defective expression of the cytoskeletal protein dystrophin (Dp427). Selected autonomic and central neurons, including retinal neurons, express Dp427 and/or dystrophin shorter isoforms. Because of this, DMD patients may also experience different forms of cognitive impairment, neurological and autonomic disorders, and specific visual defects. DMD-related damages to the nervous system are established during development, suggesting a role for all dystrophin isoforms in neural circuit development and differentiation; however, to date, their function in retinogenesis has never been investigated. In this large-scale study, we analyzed whether the lack of Dp427 affects late retinogenesis in the *mdx* mouse, the most well studied animal model of DMD. Retinal gene expression and layer maturation, as well as neural cell proliferation, apoptosis, and differentiation, were evaluated in E18 and/or P0, P5, P10, and adult mice. In *mdx* mice, expression of *Capn3*, *Id3* (E18-P5), and *Dtnb* (P5) genes, encoding proteins involved in different aspects of retina development and synaptogenesis (e.g., Calpain 3, DNA-binding protein inhibitor-3, and β-dystrobrevin, respectively), was transiently reduced compared to age-matched wild type mice. Concomitantly, a difference in the time required for the retinal ganglion cell layer to reach appropriate thickness was observed (P0–P5). Immunolabeling for specific cell markers also evidenced a significant dysregulation in the number of GABAergic amacrine cells (P5–P10), a transient decrease in the area immunopositive for the Vesicular Glutamate Transporter 1 (VGluT1) during ribbon synapse maturation (P10) and a reduction in the number of calretinin^+^ retinal ganglion cells (RGCs) (adults). Finally, the number of proliferating retinal progenitor cells (P5–P10) and apoptotic cells (P10) was reduced. These results support the hypothesis of a role for Dp427 during late retinogenesis different from those proposed in consolidated neural circuits. In particular, Dp427 may be involved in shaping specific steps of retina differentiation. Notably, although most of the above described quantitative alterations recover over time, the number of calretinin^+^ RGCs is reduced only in the mature retina. This suggests that alterations subtler than the timing of retinal maturation may occur, a hypothesis that demands further in-depth functional studies.

## Introduction

Duchenne muscular dystrophy (DMD) is a severe X-linked myodegenerative disease caused by defective expression of full-length dystrophin, a cortical cytoskeletal protein with a molecular mass of 427 kDa (Dp427) (Koenig et al., 1987; Blake et al., 2002; Mercuri et al., 2019). The DMD gene also encodes a number of shorter dystrophin isoforms, named accordingly to their molecular mass (Dp260, Dp140, Dp116, and Dp7), which are transcribed because of independent internal promoter activities and/or alternative splicing (Blake et al., 1999, 2002). Within cells, Dp427 and its short isoforms are associated with a large glycoproteic complex, the central core of which is dystroglycan (DG). One main function of the dystrophin-DG complex (DGC) is to bridge extracellular matrix (ECM) proteins to the cortical actin cytoskeleton, a connection which mechanically stabilizes cytoskeletal proteins, maintains in place signaling molecules, and protects the plasma membrane from ruptures (Davies and Nowak, 2006). Although this role is well established in muscles, where Dp427 is highly expressed and is the only isoform present, other and more diversified functions have been highlighted in different cell types, such as specific populations of autonomic, brain, and retina neurons, which may also express short isoforms (De Stefano et al., 1997; Wersinger et al., 2011; Waite et al., 2012). In the mature nervous system of both humans and rodents, Dp427, some of its short isoforms (Dp260, Dp140, Dp71), and DGC components have been described in association to GABAergic (Knuesel et al., 1999; Vaillend and Billard, 2002; Vaillend and Chaussenot, 2017), cholinergic (Zaccaria et al., 2000; Di Angelantonio et al., 2011), and glutamatergic (Miranda et al., 2011) synapses, along axons and within growth cones (Lombardi et al., 2017). Because of this diversified localization, it is generally believed that dystrophins/DGC contribute, more or less directly, to the stabilization of ionic channels (Gee et al., 1998; Connors et al., 2004; Leonoudakis et al., 2004), neurotransmitter receptors (Knuesel et al., 1999), neurotrophic factor receptors (Lombardi et al., 2008, 2017), and proteins involved in intracellular signaling pathways (Spence et al., 2004; Constantin, 2014; Lombardi et al., 2017; Fragapane et al., 2020).

In DMD, lack of Dp427 is the cause of a progressive degeneration and physiological impairment of all muscle types (Chu et al., 2002; Wallace and McNally, 2009); however, DMD patients also experience a high incidence of significant neurological disorders (Mehler, 2000; Anderson et al., 2002; Cyrulnik and Hinton, 2008; Hinton et al., 2009; Waite et al., 2009; Ricotti et al., 2016b), the severity of which depends on the type, number, and location of mutations within the DMD gene (Doorenweerd et al., 2017). In addition, the more severe the pathology, the more pronounced is the onset of visual defects (Ricotti et al., 2016a), such as impaired red-green color vision (Costa et al., 2007), loss of contrast sensitivity (Costa et al., 2011; Barboni et al., 2013), negative scotopic electroretinogram (ERG), and unbalanced a-b wave amplitude ratios (Cibis et al., 1993; Pillers et al., 1993, 1999; Fitzgerald et al., 1994; Sigesmund et al., 1994). This abnormal signal transmission is generated at the level of both rod and cone photoreceptors and their post-synaptic ON bipolar cells, in the outer plexiform layer (OPL) of the retina (Stockton and Slaughter, 1989; Fitzgerald et al., 1994; Barboni et al., 2013). Supporting this hypothesis, first immunohistochemical and ultrastructural studies on the retina of different species, including human, localized the dystrophin isoform Dp260 and DGC components in pre-synaptic terminals of photoreceptors (Drenckhahn et al., 1996; Schmitz and Drenckhahn, 1997; Ueda et al., 1997a, b; Koulen et al., 1998; Blank et al., 1999; Jastrow et al., 2006; Omori et al., 2012). However, in more recent years, a revision of dystrophin isoforms (Dp427, Dp260, Dp140, and Dp71) localization in the mouse retina has demonstrated that Dp427 is expressed in both outer nuclear layer (ONL), along with the most abundant Dp260 and Dp140, and inner nuclear layer (INL), where the other two isoforms are less represented. In particular, both Dp427 mRNA and protein were detected within ON bipolar cells, some amacrine cells (ACs), and, possibly, OFF bipolar cells (Wersinger et al., 2011).

The *mdx* mouse, the most well studied animal model of DMD, which lacks the sole Dp427 isoform, also displays specific neurophysiological abnormalities (Coccurello et al., 2002; Vaillend et al., 2004; Chaussenot et al., 2015; Miranda et al., 2015; Vaillend and Chaussenot, 2017). These, however, are milder compared to those described in DMD patients, due to the unaffected expression of all other dystrophin isoforms, which are considered more crucial than the Dp427 in brain physiopathology. As a matter of fact, some brain functions are decreased in animal models as the *mdx^3*Cv*^* mouse (X linked muscular dystrophy 3, Verne Chapman), a *mdx* mouse variant generated with *N*-ethylnitrosourea chemical mutagenesis and carrying a point mutation in intron 65 of the DMD gene, which abrogates the expression of all shorter dystrophin isoforms (Im et al., 1996; Willmann et al., 2009). Similar considerations can be drawn for the aforementioned visual defects (Tsai et al., 2016), which are either absent or less severe in *mdx* mice compared to DMD patients and *mdx^3*Cv*^* mice (Costa et al., 2007; Costa et al., 2011; Tsai et al., 2016; Bucher et al., 2019). However, upstream to all these considerations is the developmental manifestation of the dystrophin isoforms in the nervous system, which can vary among species, and their co-expression with genes implicated in neurodevelopmental disorders (Doorenweerd et al., 2017). Therefore, DMD can also be considered as a neurodevelopmental disease, since nervous system alterations are established before birth and apparently do not progress after it. Nevertheless, a delay in post-natal development during the first 4 years of life, and a pronounced cerebral atrophy in adult individuals, were reported (Yoshioka et al., 1980; Pane et al., 2013).

Although Dp260 and Dp140 apparently play a prominent functional role in the adult retina, early steps in retinal development and differentiation could mainly depend on Dp427. This idea stems from studies on rodent retinas reporting a significant pre-natal expression of Dp427 alone (Rodius et al., 1997; Bucher et al., 2019). Accordingly, studies on the autonomic and central nervous system of *mdx* mice reported defects in axonal growth and regeneration (Zaccaria et al., 2000; Lombardi et al., 2008, 2017), in adult hippocampal neurogenesis (Deng et al., 2009), and in neuronal survival, migration, and differentiation (Sbriccoli et al., 1995; Carretta et al., 2001; De Stefano et al., 2005; Lombardi et al., 2008).

To date, it is not known whether full length dystrophin, despite its minor role in adult retina physiology compared to Dp260 and Dp140 + Dp71, may have some specific function in retinal development, when it is the more abundant, if not the only, isoform expressed. In this large-scale study we analyzed whether the lack of Dp427 would affect some aspects of late retinogenesis, by analyzing retinal gene expression, layer organization, neural stem cell proliferation, apoptosis, and differentiation.

## Materials and Methods

### Animals

Wild type (C57BL/10) and genetically dystrophic (C57BL/10ScSn-Dmdmdx/J) mice (The Jackson Laboratory, Bar Harbor, ME, United States) of embryonic day 18 (E18), postnatal days 0 (P0), 5 (P5), 10 (P10), and 6–7 weeks were used. At pre- (E18) and early post-natal stages (P0–P10), the majority of the retinal cell types initiate and complete their differentiation, outnumbered cells die by apoptosis and the retinal layers are formed. In mice, this cohort of events, to which we refer as “late retinogenesis,” occurs at stages in mice that correspond to developmental weeks 14–28 of the human retina (Aldiri et al., 2017). The *mdx* mouse is the most widely used animal model for DMD, characterized by a naturally occurring nonsense point mutation (C-to-T transition) in exon 23, which aborts full-length dystrophin expression, but preserves that of short dystrophin isoforms (Sicinski et al., 1989). This model is characterized by the typical muscular degeneration, although milder than humans as occurring in waves, and diversified brain abnormalities (Anderson et al., 2002).

All studies were carried out in accordance with The Code of Ethics of the EU Directive 2010/63/EU. All efforts were made to minimize animal suffering, reduce the number of animals used, and utilize alternatives to *in vivo* techniques. The experimental procedures and protocols were approved by the Ethical Committee for Animal Research of the Italian Ministry of Public Health. Mice were housed in cages (maximum five per cage) and were maintained on a 12 h light-dark cycle with free access to food and water.

### Paraffin Embedding and Morphometric Analysis

P0 (*n* = 4), P5 (*n* = 3), P10 (*n* = 3), and 6–7-week-old (*n* = 3) wild type and *mdx* mice were deeply anesthetized with isoflurane and killed by decapitation. Mice used for each time point were obtained from different litters. Eyes were quickly removed on ice, fixed by immersion, for 48 h at 4°C, in 2% paraformaldehyde and 1.5% glutaraldehyde in 0.1 M phosphate buffer (PB), pH 7.4, and then paraffin embedded. Briefly, eyes were dehydrated at RT through an ascending series of ethyl alcohols (50%, for 30 min, 70%, for 30 min, 80%, for 30 min, 95%, for 30 min, and 100%, for 1 h), cleared in benzene (30 min at RT), impregnated in a solution 1:1 of benzene and paraffin (30 min at 60°C), followed by 3 × 30 min steps in pure paraffin, at 60°C, and finally embedded in solid paraffin blocks. Eyes were cut, along a nasal-temporal (N-T) horizontal plane, at an LKB Bromma Rotary-One microtome (LKB, Bromma, Sweden) in 10 μm-thick sections, collected on Menzel Superfrost Charged Slides (Menzel, Braunschweig, Germany). After rehydration through a series of descending ethyl alcohols (100, 70, and 50%) and distilled water, sections were stained (3 min at RT) in a 0.5% solution of cresyl violet, rinsed in distilled water, dehydrated with a series of ascending ethyl alcohol (50, 70, 80, and 100%) and xylene, let dry, and coverslipped with Eukitt mounting medium. Sections were viewed at an AxioScop2 light microscope (Carl Zeiss, Jena, Germany), equipped with an AxioCam MRc5 video camera, photographed at a 63x magnification and processed with the AxioVision 4.8.2 SP3 software.

Thickness of both outer segment (OS) and inner segment (IS) of photoreceptors, ONL, OPL, INL, inner plexiform layer (IPL), and ganglion cell layer (GCL) was measured, by using the Image J software. Measurements were taken at the posterior part of the eye bulb, on sections where the exit of the optic nerve was visible. More specifically, two pictures were taken at both sides of the optic nerve exit; the distance of the photographic field from the optic nerve stalk varied depending on the size of eye, ranging from 20 to 100 μm. Four to six serial sections, 100 μm apart, were chosen, for a total of 8–12 photographic field/eye. This region is the same that will be taken into consideration for the following quantitative analyses on sections immunolabeled for the different cell specific markers.

### Immunofluorescence

P0 (*n* = 3–4), P5 (*n* = 3–5), P10 (*n* = 3–4), and 6–7-week-old (*n* = 3–4) wild type and *mdx* mice were deeply anesthetized with isoflurane and killed by decapitation. Eye were quickly removed on ice and fixed by immersion, for 48 h at 4°C, in 4% paraformaldehyde and 4% sucrose in 0.1M PB. After a 48 h cryoprotection in 30% sucrose in 0.9% NaCl, eyes were embedded in OCT compound, quickly frozen and cut, along a N-T horizontal plane, at a Leica cryostat (Leica Microsystems, Wetzlar, Germany) in 10 μm-thick sections, collected on charged glass slides. To block antibody non-specific binding sites, sections were incubated, for 1 h at RT, in 1% Bovine Serum Albumin (BSA), 10% normal goat serum (NGS), and 0.5% Triton X-100 in phosphate buffered saline (PBS), and then, overnight, at 4°C, with one of the primary antibodies (listed in Table 1) diluted in 1% BSA, 1% NGS, and 0.2% Triton X-100 in PBS. An antigen retrieval step was performed before the glutamine synthetase immunostaining; briefly, sections were incubated (25 min at 60°C) in 10 mM sodium citrate and 0.05% Tween 20 (pH 6), rinsed in the same solution at RT and afterward in PBS, for 5 min. Following primary antibody incubation, sections were rinsed (3 × 10 min) in PBS, incubated with both the Cy3-conjugated goat anti-rabbit IgG secondary antibody (Jackson Immunoresearch Laboratories, Inc., West Grove, PA, United States) and the Hoechst nuclear staining (Invitrogen, Ltd., Inchinnan Business Park, Paisley, United Kingdom), diluted 1:1000 and 1:40000, respectively, in 1% BSA, 1% NGS, and 0.2% Triton X-100 in PBS, for 1 h at RT. After a rinse (3 × 10 min) in PBS, sections were coverslipped with the ProLong Gold Antifade Reagent (Invitrogen). Negative controls were obtained by omitting the primary antibody. Sections were viewed and photographed at 40x, 63x immersion-oil, and 100x immersion-oil magnifications at a ZEISS AxioScop2 light microscope. To optimize image resolution, each field was first photographed at the level of best focus plane, then two other images were taken one step above and below this plane, and finally the three pictures were merged together by using the ImageJ Software.

**TABLE 1:** Primary antibodies used for immunofluorescence.

Antibody	Dilution	Company
Rabbit anti-PKC-α (BC)	1:300	Abcam (Cambridge, United Kingdom)
Rabbit anti-VGluT1 (ribbon-synapses)	1:500	Abcam
Rabbit anti-GABA (GABAergic AC)	1:80	Abcam
Rabbit anti-Glutamine Synthetase (Müller cell)	1:100	Abcam
Rabbit anti-Cleaved Caspase-3 (apoptotic cell)	1:300	Cell Signaling (Beverly, MA, United States)
Rabbit anti-calbindin (HC)	1:1000	Merck Millipore (Billerica, MA, United States)
Rabbit anti-recoverin (photoreceptors)	1:1000	Merck Millipore
Rabbit anti-Tyrosine Hydroxylase (dopaminergic AC)	1:100	Merck Millipore
Rabbit anti-calretinin (RGC)	1:500	Swant (Marly, Switzerland)

Recoverin and VGluT1 immunopositive cells were viewed at a ZEISS LSM780 confocal microscope, using a 63x immersion-oil objective. Each section was scanned in 0.5 μm thick optical sections; the series range was determined by setting the upper and the lower thresholds with the Z/Y position for spatial image series setting. Final images are shown as a transparency of all layers merged together.

### EdU Cell Proliferation Assay

Mice were intraperitoneally injected with 25 mg/kg of 5-ethynyl-2′-deoxyuridine (EdU), a thymidine analog optimal for labeling proliferating cells in the nervous system (Chehrehasa et al., 2009; Zeng et al., 2010). P5 (*n* = 5–6) and P10 (*n* = 3–4) mice were injected 1 day before sacrifice (P4 and P9, respectively); differently, proliferating cells in P0 mice were revealed by injecting pregnant mothers (*n* = 3) at the 18th day of gestation. For each time point, mice were obtained from different litters. Mice were deeply anesthetized with isoflurane and killed by decapitation. Eye were quickly removed on ice, embedded in OCT compound without fixation, frozen and cut, along a N-T horizontal plane, at a Leica cryostat into 10 μm-thick sections, collected on charged glass slides. EdU labeling was revealed by using the Click-IT^TM^ EdU Cell Proliferation Kit for Imaging, Alexa Fluor^TM^ 488 dye (Invitrogen, cat. n. C10337), according to manufacturer’s instructions. Briefly, sections were fixed with 4% paraformaldehyde in PBS (10 min at RT), rinsed in PBS, permeabilized with 0.5% Triton X-100 in PBS (20 min at RT), rinsed again, and then incubated with the Click-IT reaction cocktail (30 min at RT). After a rinse with 3% BSA in PBS, sections were incubated with Hoechst nuclear stain, rinsed in PBS, and coverslipped with the ProLong Gold Antifade Reagent. EDU^+^ cells were viewed under a ZEISS LSM780 confocal microscope using a 63x immersion-oil objective, as previously described.

### Quantitative Analysis

Quantitative analysis of immunofluorescent cells was carried out on pictures taken with 40x or 63x objectives, depending on the type of cell analyzed. The images were taken in the posterior part of the retina, at the level of the optic nerve exit. A variable number of serial sections were photographed for each eye, depending on the size of the eye, the plane of cut, and the quality of the sections: P0, 3–6 sections; P5, 4–11 sections; P10, 3–11 sections; 6–7 weeks, 5–10 sections. However, chosen sections were at least 100 μm apart, which allowed us to avoid the risk of counting the same cell twice. One picture was taken at each side of the optic nerve exit, the linear length of the framed portion of the retina was measured, and only immunopositive cells bearing a clear nuclear staining were counted. Total cell numbers were normalized to 1 mm of linear length. Differently, due to their scattered distribution, the number of apoptotic cells, identified as immunopositive for cleaved caspase-3 (CC-3), were counted along the entire perimeter of the retina of each section.

Quantification of cell structures immunopositive for recoverin and VGluT1 was, instead, performed by measuring the mean fluorescence intensity (MFI) and the total fluorescent area (FA)/a region of interest (ROI), using the ImageJ software.

Quantitative analysis of EdU^+^ cells was made on confocal images taken along the entire length of the retina, dividing the counted cells into three categories: cells counted in the posterior (close to the optic nerve), middle, and anterior part (close to the ciliary body) of the eye. Eye sections for quantitative analysis were chosen at the level of optic nerve exit; for each image, EdU^+^ cells were counted within a selected ROI and final cell number was normalized to 40,000 μm^2^.

### RT-PCR and Real Time RT-PCR

Pools of E18 mouse retinas were used to verify the pre-natal expression of Dp427 in wild type mice. At this aim, RT-PCR with primer pairs specific for wild type and mutant dp427 was performed using the Qiagen One-Step RT-PCR kit and total RNA purified as described below, according to manufacturer instructions. After 30 PCR cycles, 20 microliters of each reaction were run in a 1.5% agarose gel. Primer sequences are reported in Supplementary Table S1 and were previously described, along with PCR cycling conditions (Rando et al., 2000).

For real time RT-PCR, E18 (*n* = 3–6), P0 (*n* = 3), P5 (*n* = 3–6), P10 (*n* = 3–6), and 6–7-week-old (*n* = 3) wild type and *mdx* mice were killed by decapitation, previous deep anesthesia with isoflurane for P0-adult mice. For each time point, mice were obtained from different litters. Eye were quickly removed on ice and retinas were gently dissected in HBSS, rapidly frozen, and stored at −80°C until use. Total RNA was isolated from frozen retinal tissues using the RNeasy Micro Kit (Qiagen, Hilden, Germany), according to manufacturer’s instructions, followed by quantification with a NanoDrop 2000 spectrophotometer (Thermo Scientific, Milan, Italy). The 260/280 and 260/230 ratios were also determined, with values usually in the range of 2, indicating clean RNA preparations. Purified total RNA was then reverse-transcribed using the Qiagen QuantiTect Reverse Transcription Kit and amplified on a Rotor-Gene Q (Qiagen), using the Qiagen QuantiFast SYBR Green PCR Kit. Primer pairs used for real time RT-PCR assays were purchased from Qiagen or designed using PRIMER3^1^ and are listed in Supplementary Table S1. Relative gene expression levels in different samples were determined as previously described (Carucci et al., 2017), using *Eef1a1* as a reference gene.

### Electrophoresis and Immunoblotting

P0, P5, and P10 wild type and *mdx* mice were deeply anesthetized with isoflurane (Merial, Milan, Italy) and killed by decapitation. Retinas were quickly removed on ice, frozen in dry ice, and stored at −80°C until use. Each retina was homogenized in 20 μl of ice-cold RIPA buffer (50 mM Tris/HCl at pH 7.6, 150 mM NaCl, 1 mM ethylenediaminetetraacetic acid [EDTA], 1% sodium dodecyl sulfate [SDS], 1% Triton X-100, 1X protease inhibitor cocktail [Sigma-Aldrich], 1 mM phenylmethylsulphonyl fluoride [PMSF], 0.2 mM Na_3_VO_4_, and 1 mM NaF) and sonicated for 30 s at 30 kHz with a UP100H Ultrasonic Processor (Dr. Hielsher GmbH, Teltow, Germany). Tissue homogenates were centrifuged at 18407 g (*r*_max_ 8.4) for 10 min at 4°C, and a measured aliquot of the supernatant was used to determine protein concentration by using the Micro BCA kit (Pierce, Rockford, IL, United States). Loading buffer (4X: 200 mM Tris/HCl pH 6.8, 4% SDS, 30% glycerol, 4% β-mercaptoethanol, and 4% blue bromophenol) was added to the homogenates up to the 1X final concentration; homogenates were, then, heated for 5 min at 95°C and kept at −20°C until use. Sixty micrograms of proteins were loaded on a 4% sodium dodecyl sulphate-polyacrylamide gel and separated by electrophoresis (SDS-PAGE). HyperPAGE Pre-stained Protein Markers (AbCam, Cambridge, United Kingdom) of precisely sized recombinant proteins (245, 180, 135, 100, 75, 63, 48, 35, 25, 20, 17, 11 kDa) were used as molecular weight standards. After electrophoresis, proteins were transferred onto a nitrocellulose membrane and proper protein transfer was verified by ponceau S staining (3% ponceau S, 30% trichloroacetic acid in distilled H_2_O) for 15 min. After a rinse in distilled H_2_O followed by TTBS (20 mM Tris/HCl, pH 7.5, 500 mM NaCl, 0.05% Tween 20), membranes were incubated, for 2 h at RT, with a non-specific binding site blocking solution made of 5% defatted dry milk (DM) in TTBS, rinsed again in TTBS, and then incubated overnight at 4°C with the mouse anti-dystrophin rod domain (Dys1) (Novocastra Laboratories, Newcastle upon Tyne, United Kingdom) primary antibody, diluted 1:10 in 3% bovine serum albumin (BSA) and 0.05% NaN_3_ in TTBS. Following another rinse in TTBS, membranes were incubated (1 h at RT) with a peroxidase-conjugated goat anti-mouse IgG secondary antibody (Promega Italia, Milan, Italy), diluted 1:15000 in 2.5% DM in TTBS. Antibody-antigen binding sites were detected by enhanced chemiluminescence (ECL) West Pico Plus Substrate (Immunological Science, Rome, Italy). The immunopositive bands were visualized by exposure of the membrane to X-OMAT film plates (Kodak).

### Statistical Analysis

Morphometric analyses conducted on paraffin sections were analyzed by two-way ANOVA test with Sidak *post hoc* correction and expressed as the mean ± standard error of the mean (SEM). Differences were considered statistically significant for *p* ≤ 0.05.

For all quantitative analyses (immunofluorescence, cell proliferation), data were analyzed by either one-way ANOVA test Tukey *post hoc* or two-way ANOVA test with Sidak *post hoc* correction, and expressed as the mean ± SEM. Differences were considered statistically significant for *p* ≤ 0.05, but expression changes with *p* < 0.1 are also highlighted in Figure 12.

Data relative to gene expression were analyzed by two tailed Student’s *t-*test and expressed as the mean relative expression levels ± SEM following normalization to wild type samples. Differences were considered statistically significant for *p* ≤ 0.05.

## Results

### Morphometric Analysis Reveals Transitory Alterations, at Early Post-natal Stages, in the Thickness of the GCL of *mdx* Mice Compared to Wild Type

**FIGURE 1:**
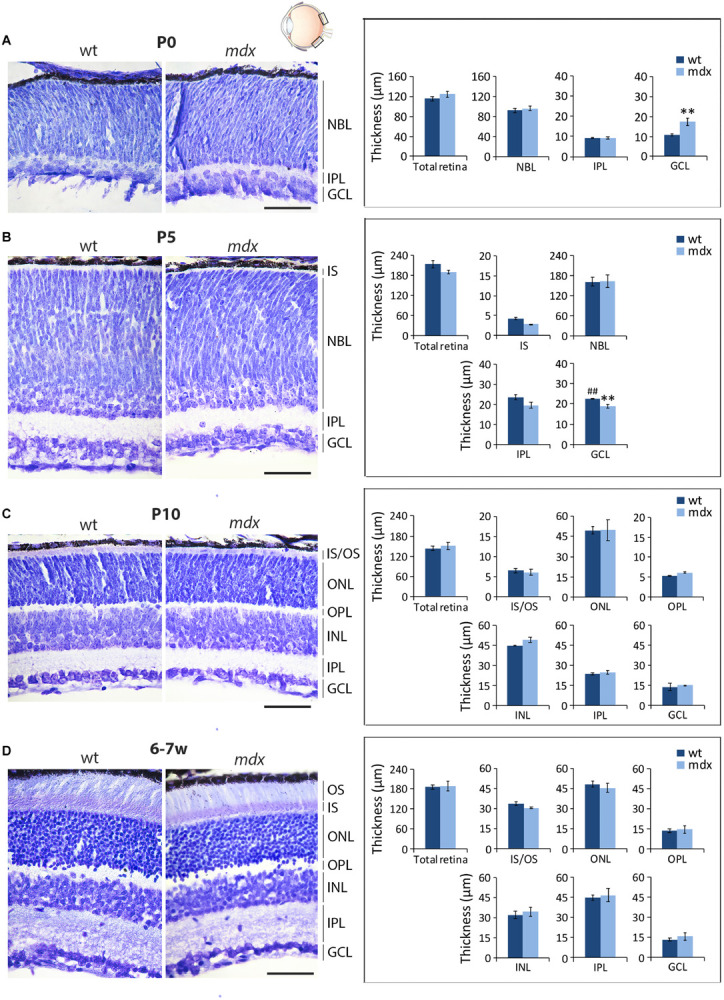
Morphometric analysis of wild type and *mdx* mouse retinas at different postnatal days reveals a transitory alteration in the thickness of the GCL of dystrophic mice. Representative cresyl violet-stained horizontal (N-T) retina paraffin sections of P0 **(A)**, P5 **(B)**, P10, **(C)** and 6–7-week-old **(D)** wild type (wt) and *mdx* mice (left), and comparative morphometric analysis of the thickness of the retinal layers (right). Measures are made in the posterior retina, at the level of the optic nerve exit (black boxes in the eye drawing). Data are analyzed by two-way ANOVA test Sidak *post hoc* and represented as the mean ± SEM. ^∗∗^*p* ≤ 0.01 (*mdx vs.* wt); ^##^p ≤ 0.01 (P5 *vs.* P0 of the same genotype). *n* = 3–4 independent experiments/postnatal date. Scale bar: 50 μm. IS, inner segment of photoreceptor; OS, outer segment of photoreceptors; NBL, neuroblastic cell layer; ONL, outer nuclear layer; OPL, outer plexiform layer; INL, inner nuclear layer; IPL, inner plexiform layer; GCL, ganglion cell layer.

In order to uncover whether the lack of Dp427 affects the gross anatomical architecture of the retina, we measured the thickness of the six main retinal layers on serial, cresyl violet-stained paraffin sections of P0, P5, P10, and 6–7-week old wild type and *mdx* mice (Figure 1, left). At birth (P0), retinal layering is still developing (Nguyen-Ba-Charvet and Chédotal, 2014; Taylor et al., 2015), and only the maturing GCL and IPL, along with a large undifferentiated neuroblastic cell layer (NBL), were evident (Figure 1A). At this stage, the GCL in *mdx* mouse retinas was significantly thicker (*p* ≤ 0.01) than that in wild type mice (Figure 1A, right). At P5, this result was reversed, as GCL thickness became significantly thinner (*p* ≤ 0.01) in the dystrophic retinas compared to wild type (Figure 1B, right), mainly because of thickening of the GCL in this genotype (Figures 1A,B, right). According to the literature (Taylor et al., 2015), at this stage, signs of separation between the INL and the ONL emerge within the NBL, and a primordium of the IS of photoreceptors could also be measured (Figure 1B, left). Despite these selective differences at P0 and P5, total retinal thickness was not different between the two genotypes (Figures 1A,B, right). Between P10 (Figure 1C, left) and 6–7 weeks (Figure 1D, left) of age, retinas of both genotypes underwent a marked maturation, with all layers progressively increasing in both thicknesses and neural cell organization. At both ages, no significant differences were seen in the thickness of the whole retina, or the single layers, between the two genotypes (Figures 1C,D, right).

### The Number of Calretinin^+^ RGCs and GABA^+^ ACs in Adult and P10 mdx Mice, Respectively, Is Significantly Reduced Compared to Wild Type

In order to establish whether the lack of Dp427 in *mdx* mice affects the progressive differentiation of the retinal cell types, we performed a quantitative analysis by immunolabeling for cell-specific markers serial, N-T horizontal retina cryosections obtained from P0 to 6–7-week old mice. Cell counts were performed in the posterior retina, at the level of the optic nerve head.

#### The Number of Cells Immunopositive for Recoverin (Photoreceptors), Calbindin (Horizontal Cells), Protein Kinase-α (Bipolar Cells), and Glutamine Synthase (Müller Cells) Is Similar Between Wild Type and mdx Mice

**FIGURE 2:**
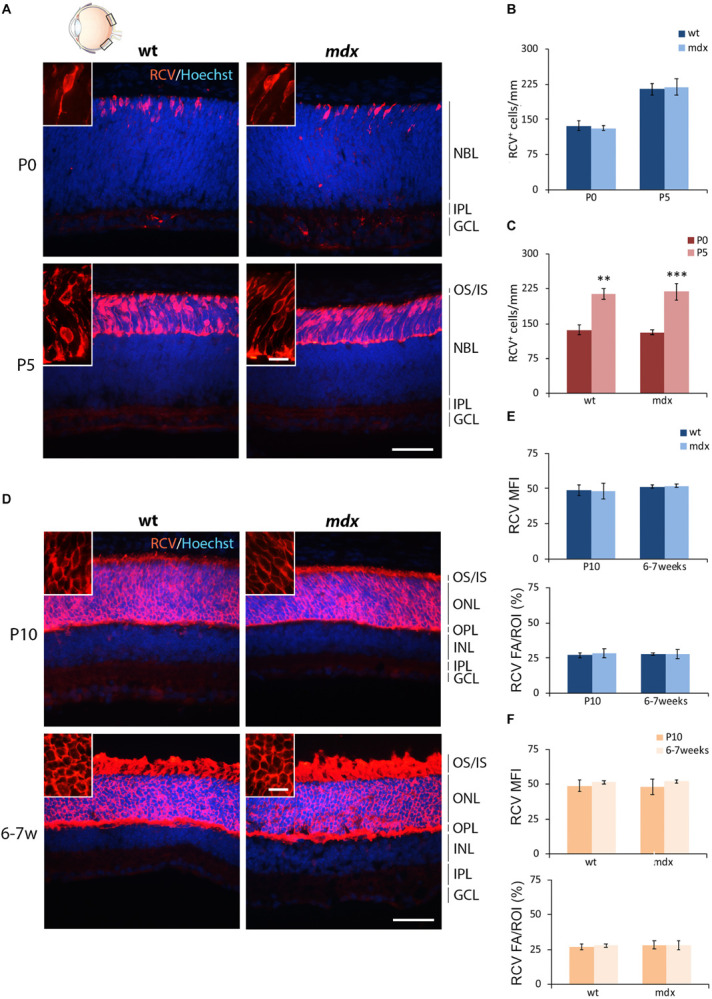
Lack of Dp427 does not alter neither the number of recoverin-immunopositive photoreceptors nor the recoverin fluorescence intensity. **(A,D)** Representative images of horizontal (N–T) retina cryosections of P0, P5, P10, and 6–7-week-old wild type (wt) and *mdx* mice immunolabeled for recoverin (RCV), a pan-photoreceptor cell marker. Nuclei are stained in blue (Hoechst). **(B,C)** Comparative quantitative analysis of the number of RCV^+^ cells/mm of retina between wt and *mdx* age-matched mice **(B)** and across post-natal days within the same genotype **(C)**. **(E,F)** Comparative quantitative analysis between wt and *mdx* age-matched mice **(E)** and across post-natal days within the same genotype **(F)** based on both mean fluorescent intensity (MFI) (normalized against the background) and percentage of RCV fluorescent area (FA)/ROI area, measured by the ImageJ software. Both cell counts **(B,C)** and measures of the fluorescence intensity **(E,F)** are made in the posterior retina (black boxes in the eye drawing). Data are analyzed by two-way ANOVA test Sidak *post hoc* and represented as the mean ± SEM. ^∗∗^*p* ≤ 0.01, ^∗∗∗^*p* ≤ 0.001 (P5 vs. P0 in **C**). *n* = 3–5 independent experiments. Scale bar: 50 μm; insets: 10 μm. OS, outer segment of photoreceptors; IS, inner segment of photoreceptors; NBL, neuroblastic cell layer; ONL, outer nuclear layer; OPL, outer plexiform layer; INL, inner nuclear layer; IPL, inner plexiform layer; GCL, ganglion cell layer.

The first order retinal neuron is represented by the rod and cone photoreceptors. Cones differentiate during embryonic development (around E14), while about 73% of the postnatal progenitor cells differentiate into rods (P0) (Young, 1985; Heavner and Pevny, 2012). For photoreceptor pan-immunolabeling, we used an antibody directed against recoverin (RCV), a Ca^2+^-binding protein (CaBP) mainly located in the OS of photoreceptors (Senin et al., 2002; Sharma et al., 2003). RCV immunofluorescence significantly increased during post-natal retinal maturation, with few cells observed at P0, most probably representing the cone population, with photoreceptors becoming progressively immunopositive throughout the following post-natal stages (Figures 2A,D). Possible differences in photoreceptor differentiation between wild type and *mdx* mice were analyzed by counting the number of RCV^+^ cells in P0 and P5 mice, when cells were distant enough to be recognized individually (Figures 2B,C). At P10 and 6–7 weeks, RCV^+^ cells were so numerous and packed together that this approach was substituted by the analysis of both RCV MFI and RCV FA/ROI ratio (Figures 2E,F). Throughout these postnatal stages, no differences between the two genotypes were observed, neither in the number of RCV^+^ cells (Figure 2B) nor in the RCV MFI and FA/ROI (Figure 2E). In addition, both genotypes showed a similar increase in the number of RCV^+^ cells between P0 and P5 (Figure 2C), whereas RCV fluorescence intensity remained stable between P10 and 6–7 weeks (Figure 2F).

**FIGURE 3:**
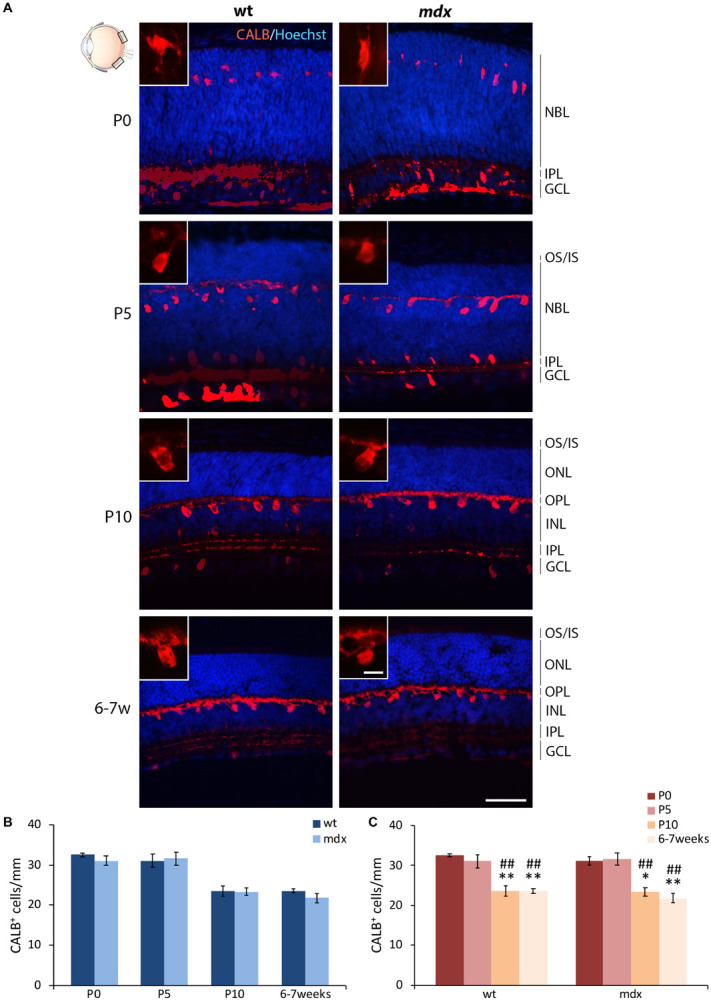
Lack of Dp427 does not alter the number of calbindin-immunopositive horizontal cells. **(A)** Representative images of horizontal (N–T) retina cryosections of P0, P5, P10, and 6–7-week-old wild type (wt) and *mdx* mice immunolabeled for calbindin (CALB). Nuclei are stained in blue (Hoechst). **(B,C)** Quantitative analysis of the number of CALB^+^ cells/mm of retina between wt and *mdx* age-matched mice **(B)** and across post-natal days within the same genotype **(C)**. Cell counts are made in the posterior retina (black boxes in the eye drawing). Data are analyzed by either two-way ANOVA test Sidak *post hoc* **(B)** or one-way ANOVA test Tukey *post hoc* **(C)** and represented as the mean ± SEM. ^∗^*p* ≤ 0.05, ^∗∗^*p* ≤ 0.01 (vs. P0); ^##^*p* ≤ 0.01 (*vs.* P5). *n* = 3–5 independent experiments. Scale bar: 50 μm; insets: 10 μm. OS, outer segment of photoreceptors; IS, inner segment of photoreceptors; NBL, neuroblastic cell layer; ONL, outer nuclear layer; OPL, outer plexiform layer; INL, inner nuclear layer; IPL, inner plexiform layer; GCL, ganglion cell layer.

Horizontal cells (HCs) differentiate between the end of embryonic life and P4–P5 (Nguyen-Ba-Charvet and Chédotal, 2014), a time at which active photoreceptors influence HC migration in the outer portion of the INL and the consequent formation of the OPL (Raven et al., 2007; Heavner and Pevny, 2012). According to this, by immunofluorescence for Calbindin (CALB), a CaBP highly expressed in HCs, we observed a progressive organization and maturation of these neurons within the appropriate retinal layers between P0 and 6–7 weeks, with no evident differences between the two genotypes (Figure 3A). Cell counting revealed no differences in the number of CALB^+^ cells between wild type and *mdx* mice, at any of the ages considered (Figure 3B). The number of immunopositive cells significantly decreased (*p* ≤ 0.01) in P10 and adult mice with respect to the P0 and P5 mice (Figure 3C), with no differences between the two genotypes.

**FIGURE 4:**
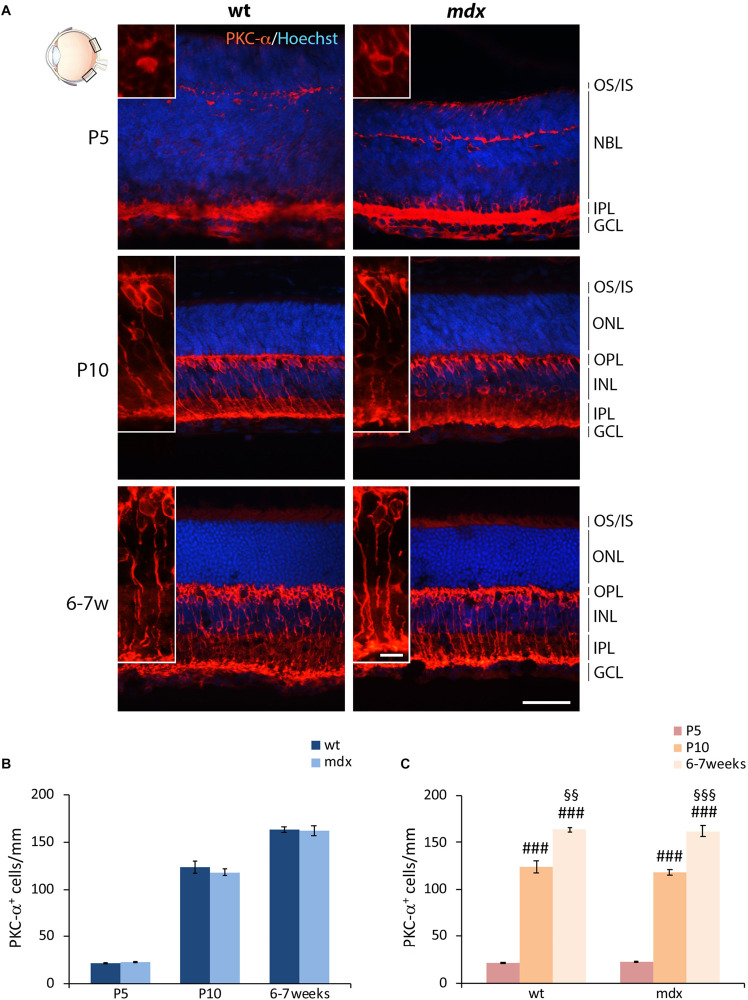
Lack of Dp427 does not alter the number of protein kinase-α-immunopositive bipolar cells. **(A)** Representative images of horizontal (N–T) retina cryosections of P5, P10, and 6–7-week-old wild type (wt) and *mdx* mice immunolabeled for PKC-α. Nuclei are stained in blue (Hoechst). **(B,C)** Comparative quantitative analysis of the number of PKC-α^+^ cells/mm of retina between wt and *mdx* age-matched mice **(B)** and across post-natal days within the same genotype **(C)**. Cell counts are made in the posterior retina (black boxes in the eye drawing). Data are analyzed by either two-way ANOVA test Sidak *post hoc* **(B)** or one-way ANOVA test Tukey *post hoc* **(C)** and represented as the mean ± SEM. *n* = 3–5 independent experiments. ^###^*p* ≤ 0.001 (*vs.* P5); ^§§^*p* ≤ 0.01, ^§§§^*p* ≤ 0.001 (*vs.* P10). Scale bar: 50 μm; insets: 10 μm. OS, outer segment of photoreceptors; IS, inner segment of photoreceptors; NBL, neuroblastic cell layer; ONL, outer nuclear layer; OPL, outer plexiform layer; INL, inner nuclear layer; IPL, inner plexiform layer; GCL, ganglion cell layer.

Bipolar cells (BCs) represent the second order retinal neurons, which are subdivided in two major subclasses, referred to as ON- and OFF-BCs. Because their full differentiation occurs after birth, we were able to perform a quantitative analysis starting by P5. The BCs marker chosen was the alpha isoform of protein kinase C (PKC-α), a Ser/Thr protein kinase mainly expressed by rod BCs (Figure 4A), although a minority of ACs and cone photoreceptors are also PKC-α^+^ (Fukuda et al., 1994). A clear pattern of differentiation from P5 to adult mice was seen by PKC-α immunolabeling; cell bodies and dendrites of mature BCs clearly localized at the INL/OPL levels, while their axons projected toward the GCL and ended within the IPL (Figure 4A). As for HCs, no gross anatomical differences were observed between the two genotypes. Likewise, quantitative analysis did not reveal differences either in the number of PKC-α^+^ between age-matched wild type and *mdx* mice (Figure 4B), or in the time course of cell maturation, which was characterized by a steady and significant increase in the number of PKC-α^+^ BCs from P5 to 6–7-week old mice (Figure 4C).

**FIGURE 5:**
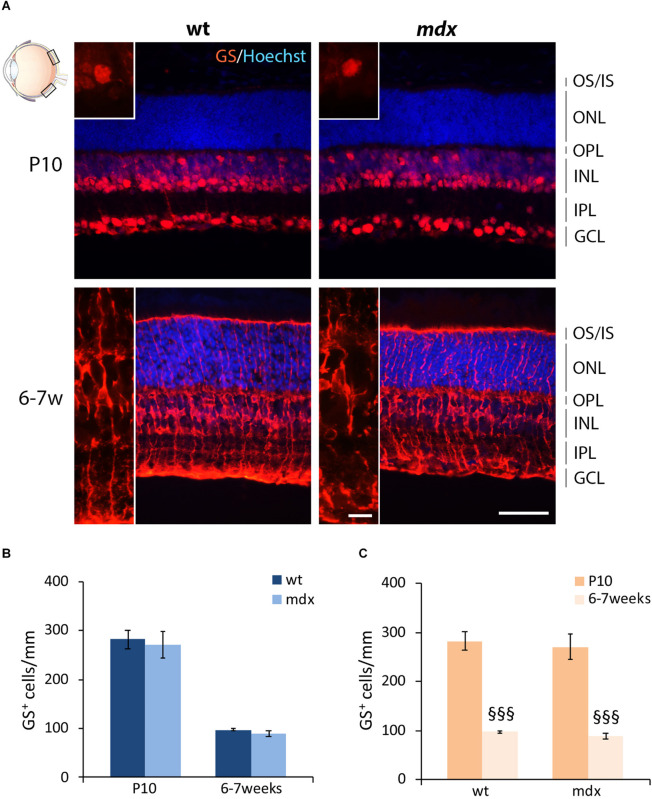
Lack of Dp427 does not alter the number of glutamine synthase-immunopositive Müller cells. **(A)** Representative images of horizontal (N–T) retina cryosections of P10 and 6–7-week-old wild type (wt) and *mdx* mice immunolabeled for glutamine synthase (GS). Nuclei are stained in blue (Hoechst). **(B,C)** Comparative quantitative analysis of the number of GS^+^ cells/mm of retina between wt and *mdx* age-matched mice **(B)** and across post-natal days within the same genotype **(C)**. Cell counts are made in the posterior retina (black boxes in the eye drawing). Data are analyzed by two-way ANOVA test Sidak *post hoc* and represented as the mean ± SEM. ^§§§^*p* ≤ 0.001 (vs. P10). *n* = 3 independent experiments. Scale bar: 50 μm; insets: 10 μm. OS, outer segment of photoreceptors; IS, inner segment of photoreceptors; ONL, outer nuclear layer; OPL, outer plexiform layer; INL, inner nuclear layer; IPL, inner plexiform layer; GCL, ganglion cell layer.

Müller cells are the predominant glial cells in the retina and one of the last cell types to differentiate (Young, 1985; Heavner and Pevny, 2012), in the second wave of retinogenesis (P0–P7), and were identified by their exclusive and intense expression of glutamine synthase (GS) (Vardimon et al., 1993). Numerous GS^+^ cell bodies were detectable from P10: one subset of small cells, clustered together, occupied the INL, while another subset of large cells, more distant from each other, were located in the GCL (Figure 5A). By 6–7 weeks, Müller cells spanned the entire retina, from the outermost portion of the ONL to the GCL (Figure 5A). No differences were observed, between the two genotypes, in both pattern of differentiation and cell distribution. Likewise, no differences were detected in the number of GS^+^ cells between age-matched wild type and *mdx* mice (Figure 5B), nor in the time course of cell number refinement within each genotype. In this case, in both wild type and *mdx* mouse retinas, GS^+^ cell bodies significantly decreased between P10 and 6–7 weeks (Figure 5C).

#### Wild Type and *mdx* Mouse Retinas Show No Major Differences in Immunofluorescence Intensity for the Vesicular Glutamate Transporter 1 at the Ribbon Synapse Level

**FIGURE 6:**
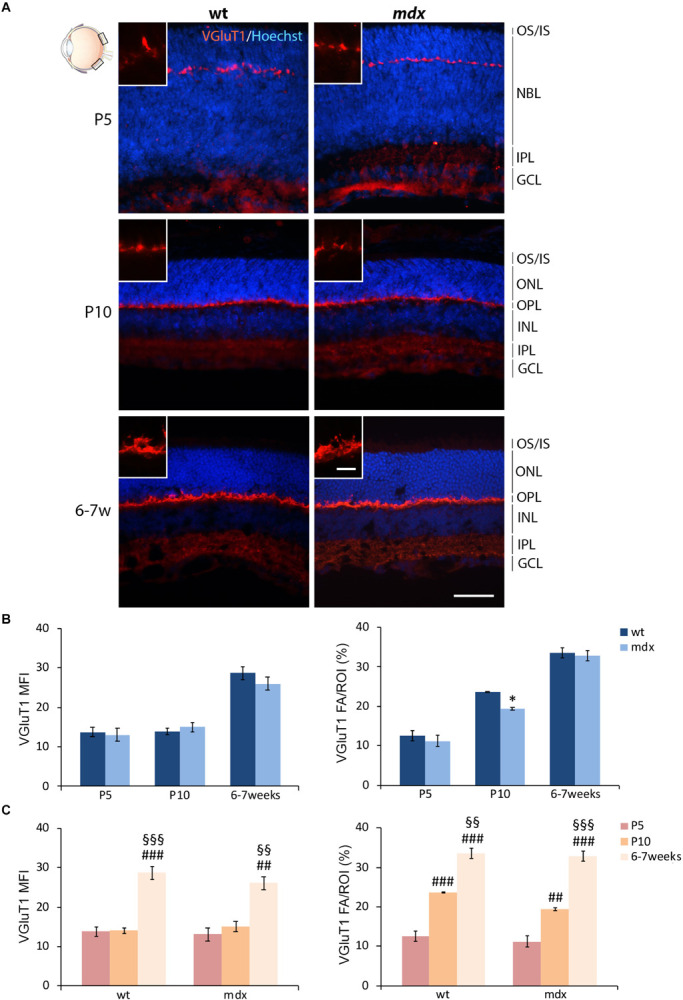
Lack of Dp427 determines a transitory reduction in the area occupied by VGluT1 immunofluorescence in P10 *mdx* mice compared to wild type. **(A)** Representative images of horizontal (N–T) retina cryosections of P5, P10, and 6–7-week-old wild type (wt) and *mdx* mice immunolabeled for the vesicular glutamate transporter 1 (VGluT1). Nuclei are stained in blue (Hoechst). **(B,C)** Comparative quantitative analyses between wt and *mdx* age-matched mice **(B)** and across post-natal days within the same genotype **(C)**. VGluT1^+^ cells are quantified as both mean fluorescent intensity (MFI) (normalized against the background) and percentage of VGluT1 fluorescent area (FA)/ROI area, measured by the Image J software. Measures were made in the posterior region of the retina (black boxes in the eye drawing). Data are analyzed by either two-way ANOVA test Sidak *post hoc* **(B)** or one-way ANOVA test Tukey *post hoc* **(C)** and represented as the mean ± SEM. ^∗^*p* ≤ 0.05 (*mdx* vs. wt); ^##^*p* ≤ 0.01, ^###^*p* ≤ 0.001 (vs. P5); ^§§^*p* ≤ 0.01, ^§§§^*p* ≤ 0.001 (vs. P10). *n* = 3 independent experiments. Scale bar: 50 μm; insets = 10 μm. OS, outer segment of photoreceptors; IS, inner segment of photoreceptors; NBL, neuroblastic cell layer; ONL, outer nuclear layer; OPL, outer plexiform layer; INL, inner nuclear layer; IPL, inner plexiform layer; GCL, ganglion cell layer.

In order to complete the analysis on the differentiation pattern of the photoreceptor-BCs binomial, the rate of maturation of ribbon synapses was evaluated by analyzing the stage-dependent changes in the intensity of immunolabeling for the vesicular glutamate transporter 1 (VGluT1) (Figure 6). As reported in the introduction, Dp427, Dp260, and DGC components are localized in the OPL in both pre- and postsynaptic compartments. Presynaptic DGC and extracellular matrix protein partners are determinant for proper ribbon synapse formation (Sato et al., 2008; Omori et al., 2012). However, because of their folded structure, immunolabeling of ribbon synapses is challenging. Therefore, this first evaluation was only intended to uncover possible gross differences in the timing of synapse formation and topographical organization.

In both wild type and *mdx* mice, VGluT1 immunopositivity was localized as a neat line of closely packed dots and structures within the OPL, clearly detectable by P5 (Figure 6A). No obvious differences were observed in the distribution of immunolabeling. Maturation of ribbon synapses was inferred by the progressive increase in both the intensity of immunolabeling and the size of the immunopositive area (Figure 6A), measured as VGluT1 MFI and FA/ROI ratio, respectively (Figures 6B,C). No statistical differences were seen in VGluT1 MFI between age-matched wild type and *mdx* mice; nonetheless, a significantly lower (*p* ≤ 0.05) VGluT1 FA/ROI ratio was recorded in P10 *mdx* mice compared to wild type (Figure 6B). Within each genotype, the VGluT1 MFI and FA/ROI ratio increased significantly from P5 toward 6–7 weeks, with a similar trend between wild type and *mdx* mice (Figure 6C). However, a more accurate study, also involving electron microscopy, will be needed to investigate these synaptic structures in detail.

#### The Number of Calretinin^+^ RGCs in the Adult *mdx* Mouse Retina Is Significantly Reduced Compared to Wild Type

**FIGURE 7:**
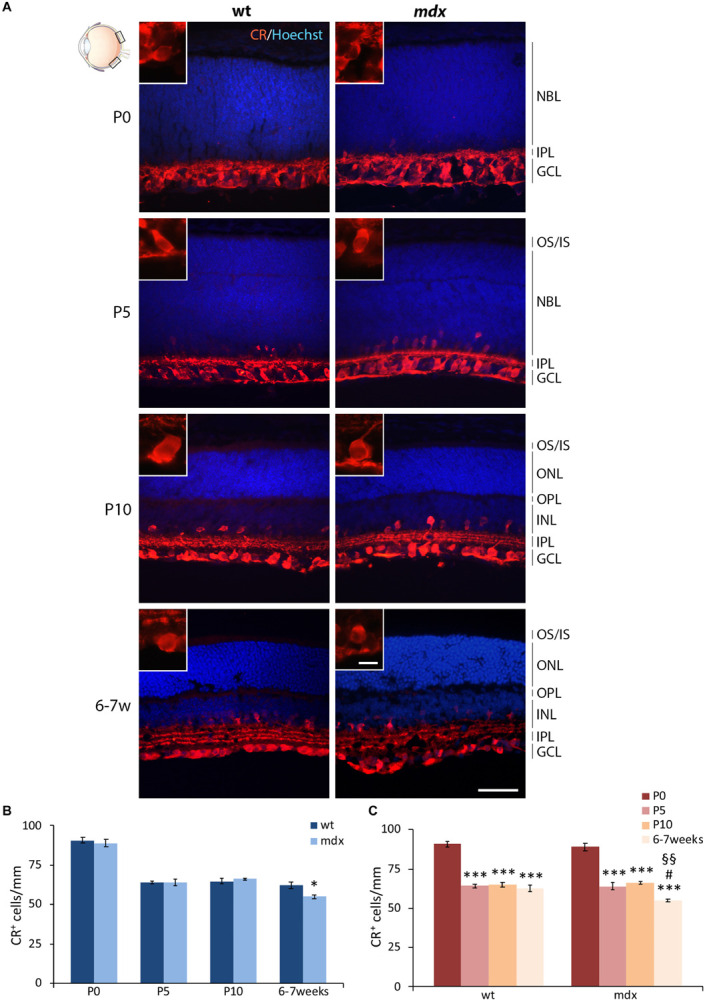
Lack of Dp427 determines a reduction in calretinin-immunopositive retinal ganglion cells in 6–7-week-old *mdx* mice. **(A)** Representative images of horizontal (N–T) retina cryosections of P0, P5, P10, and 6–7-week-old wild type (wt) and *mdx* mice immunolabeled for calretinin (CR). Nuclei are stained in blue (Hoechst). **(B,C)** Comparative quantitative analysis of the number of CR^+^ cells/mm of retina between wt and *mdx* age-matched mice **(B)** and across post-natal days within the same genotype **(C)**. Cell counts are made in the posterior retina (black boxes in the eye drawing). Data are analyzed by either two-way ANOVA test Sidak *post hoc* **(B)** or one-way ANOVA test Tukey *post hoc* **(C)** and represented as the mean ± SEM. ^∗^*p* ≤ 0.05 (*mdx* vs. wt), ^∗∗∗^*p* ≤ 0.001 (vs. P0); ^#^*p* ≤ 0.05 (vs. P5); ^§§^*p* ≤ 0.01 (vs. P10). *n* = 3–5 independent experiments. Scale bar: 50 μm; insets: 10 μm. OS, outer segment of photoreceptors; IS, inner segment of photoreceptors; NBL, neuroblastic cell layer; ONL, outer nuclear layer; OPL, outer plexiform layer; INL, inner nuclear layer; IPL, inner plexiform layer; GCL, ganglion cell layer.

Mouse RGCs are subdivided in about 40 subtypes, diversified by either functional properties or transcriptomics (Laboissonniere et al., 2019). This diversification is also characterized by the expression of a number of cell markers, among which three CaBPs: calretinin (CR), parvalbumin, and CALB (Lee et al., 2019; Kovács-Öller et al., 2020). Nearly all RGCs express CaBPs, alone or in combination; however, for this first quantitative analysis, we chose CR as the representative marker (Figure 7), based on its stronger intensity of immunolabeling and higher number of CR^+^ cells described in the dorso-central area of the retina (Kovács-Öller et al., 2020). CR immunolabeling neatly decorated the innermost layer of the retina, with both cell size and layered organization of immunopositive cell bodies and fibers increasing throughout ages (Figure 7A). Quantitative analysis revealed a significant reduction (*p* ≤ 0.05) in the number of CR^+^ cells in the GCL of 6–7-week-old *mdx* mice, compared to wild type, although no differences were observed throughout the early post-natal period (Figure 7B). Between P0 and P10, the number of CR^+^ cells changed accordingly in wild type and *mdx* mice, as the number of immunopositive cells counted at birth (P0) decreased significantly (*p* ≤ 0.001) by P5 and remained stable at P10. Notably, although the amount of CR^+^ cells was similar in P10 and adult wild type retinas, it decreased significantly (*p* ≤ 0.05) in adult *mdx* mice compared to both wild type adult and P10 *mdx* mice (Figures 7B,C).

#### The Number of GABAergic ACs in P5 and P10 *mdx* Mice Is Significantly, but Transiently, Deregulated Compared to Wild Type

In the mammalian retina ACs can be subdivided in at least 28 subtypes, based on their morphologies, sublaminar position, and physiological properties (MacNeil and Masland, 1998; Masland, 2001). In spite of their diversified neurochemical signature, the major neurotransmitter used by ACs is GABA (Brecha et al., 1988; Kosaka et al., 1988; Vaney, 1990; Masland, 2001). ACs are normally distributed within the INL, although an interesting class of GABAergic “starburst” ACs (SACs) was identified displaced in the GCL (Pérez De Sevilla Müller et al., 2007; Taylor and Smith, 2012).

**FIGURE 8:**
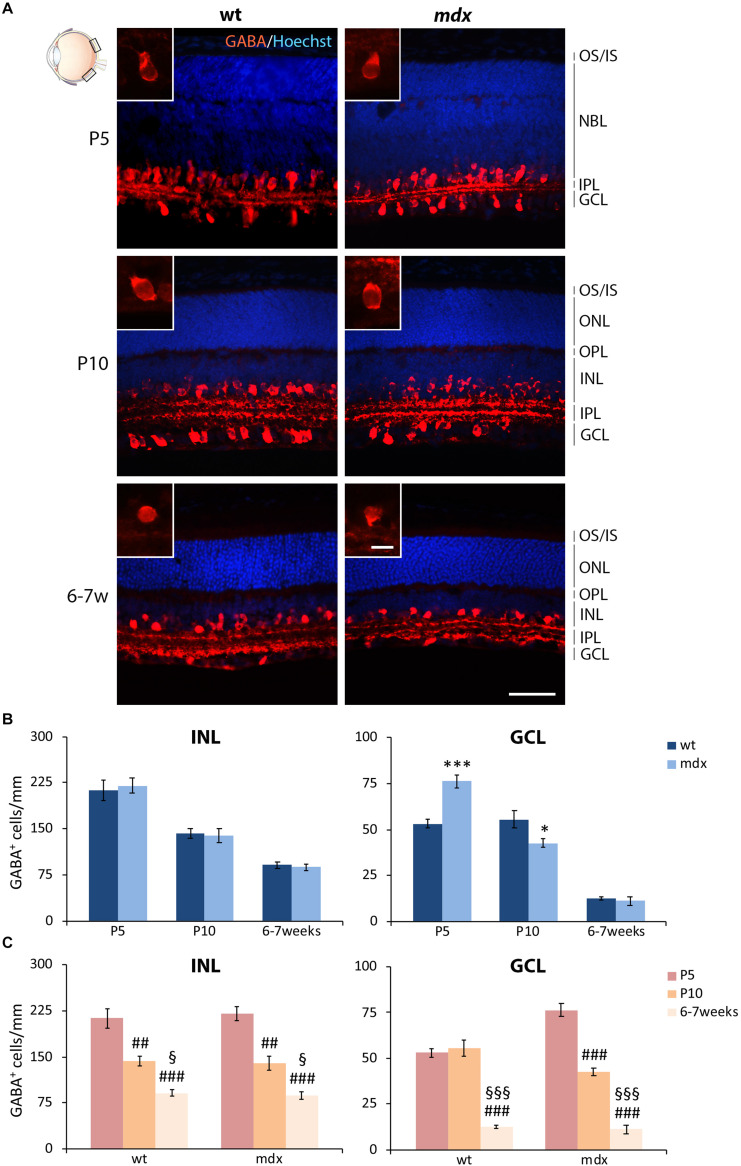
Lack of Dp427 determines transitory alterations in the number of GABA-immunopositive amacrine cells in P5 and P10 *mdx* mice compared to wild type. **(A)** Representative images of horizontal (N–T) retina cryosections of P5, P10, and 6–7-week-old wild type (wt) and *mdx* mice immunolabeled for GABA. Nuclei are stained in blue (Hoechst). **(B,C)** Comparative quantitative analysis of the number of GABA^+^ cells/mm of retina between wt and *mdx* age-matched mice **(B)** and across post-natal days within the same genotype **(C)**. The counts of immunopositive amacrine cells residing in the INL and of those located in the GCL have been kept separate. Counts are made in the posterior region of the retina (black boxes in the eye drawing). Data are analyzed by either two-way ANOVA test Sidak *post hoc* **(B)** or one-way ANOVA test Tukey *post hoc* **(C)** and represented as the mean ± SEM. ^∗^*p* ≤ 0.05, ^∗∗∗^*p* ≤ 0.001 (*mdx* vs. wt); ^##^*p* ≤ 0.01, ^###^*p* ≤ 0.001 (vs. P5); ^§^*p* ≤ 0.05, ^§§§^*p* ≤ 0.001 (vs. P10). *n* = 3–5 independent experiments. Scale bar: 50 μm; insets: 10 μm. OS, outer segment of photoreceptors; IS, inner segment of photoreceptors; NBL, neuroblastic cell layer; ONL, outer nuclear layer; OPL, outer plexiform layer; INL, inner nuclear layer; IPL, inner plexiform layer; GCL, ganglion cell layer.

In this study, we focused the quantitative analysis of ACs on both the most represented GABAergic phenotype and the dopaminergic phenotype. Numerous GABA^+^ ACs were clearly detectable by P5, distributed between the INL and GCL, a subdivision which became clearer during the following stages of retinogenesis (Figure 8A). At P10, and more clearly at 6–7 weeks, the prominent dendritic arborization sharply stratified within the ON and OFF sublaminae of the IPL (Figure 8A). Although this precise organization was present in both wild type and *mdx* mice, a quantitative analysis conducted separately for the ACs localized in the GCL and those placed in the INL uncovered a discrepancy between the two genotypes in the number of displaced GABA^+^ ACs located in the GCL at P5 and P10 (Figure 8B). More specifically, in P5 *mdx* mice, the number of GABA^+^ cells was significantly higher (*p* ≤ 0.001) compared to wild type, and became significantly (*p* ≤ 0.05) lower at P10 (Figure 8B). The number of GABA^+^ ACs in the INL, instead, did not differ between age-matched mice of the two genotypes (Figure 8B). Postnatal changes in AC number within each genotype were also similar and showed a progressive significant decrease in the number of GABA^+^ cells, in both INL and GCL. In *mdx* mice, however, ACs in the GCL began to decrease earlier than in wild type mice, possibly due to the increased number of ACs detected in the GCL at P5 (Figure 8C).

Dopaminergic ACs are interplexiform neurons with multiple modulatory effects in different species (Jensen and Daw, 1984; Piccolino et al., 1987; Gustincich et al., 1997). We quantified this type of ACs by immunolabeling for tyrosine hydroxylase (TH), the rate limiting enzyme for catecholamine synthesis. Dopaminergic ACs are not numerous (26 ± 5.7 cells/mm^2^) (Gustincich et al., 1997) and in our cryosections appeared as single units in the INL, starting by P10. Therefore, their number calculated *per* linear mm was extremely low; however, no differences were observed between wild type and *mdx* mice (not shown).

### The Number of Proliferating Retinal Progenitor Cells in P5 and P10 *mdx* Mice Is Reduced Compared to Age-Matched Wild Type Mice

**FIGURE 9:**
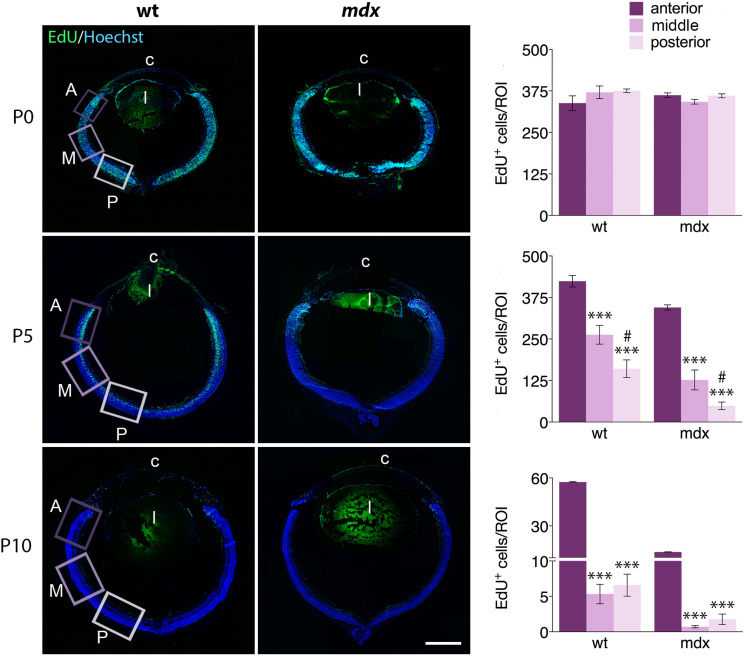
The pattern of distribution of EdU^+^ proliferative cells along the retinal perimeter does not differ between wild type and *mdx* mice. On the left side of the figure, representative images of EdU^+^ cell (green) distribution along the entire perimeter of horizontal (N–T) retina cryosections of P0, P5, and P10 wild type (wt) and *mdx* mice. Nuclei are stained in blue (Hoechst); c: cornea; l: lens. On the right side, comparative quantitative analysis of the number of EdU^+^ proliferating cells/region of interest (ROI), distinct by regions: anterior, middle and posterior retina. Data are analyzed by one-way ANOVA test Tukey *post hoc* and represented as the mean ± SEM. ^∗∗∗^*p* ≤ 0.001 (vs. anterior region); ^#^*p* ≤ 0.05 (vs. middle region). *n* = 3–6 independent experiments. Scale bar: 200 μm.

In *mdx* mice, lack of Dp427 affects differentiation, migration, and survival of several central and autonomic neuronal populations (Sbriccoli et al., 1995; Carretta et al., 2001; De Stefano et al., 2005; Lombardi et al., 2008) and induce a deregulation of the proliferation/differentiation ratio in adult hippocampal neurogenesis (Deng et al., 2009). To understand whether the lack of Dp427 could also affect proliferation and migration of retinal progenitor cells (RPCs), we quantified the number of dividing cells, labeled with AlexaFluor-tagged EdU, in anterior (A, close to the ciliary body), middle (M), and posterior (P) regions of the retina, performing this analysis in sections cut at the level of the optic nerve head. At first, we analyzed the relative distribution of EdU^+^ cells within the three selected regions in P0, P5, and P10 mice, finding a similar pattern in the two genotypes (Figure 9). In particular, at P0, the number of EdU^+^ cells/ROI, which largely occupy the NBL, was similar throughout the three regions. At P5, this number significantly (*p* ≤ 0.001) and progressively decreased from the A region toward the most posteriors ones (A > M > P). The decrease of EdU^+^ cells between M and P regions was also significant (*p* ≤ 0.05). P10 mouse retinas were characterized by a drastic decrease in the number of proliferating cells along the entire retina; the number of EdU^+^ cells in the A region was still significantly higher (*p* > 0.001) compared to the M and P regions, while no differences were observed between these two in each genotype (Figure 9).

**FIGURE 10:**
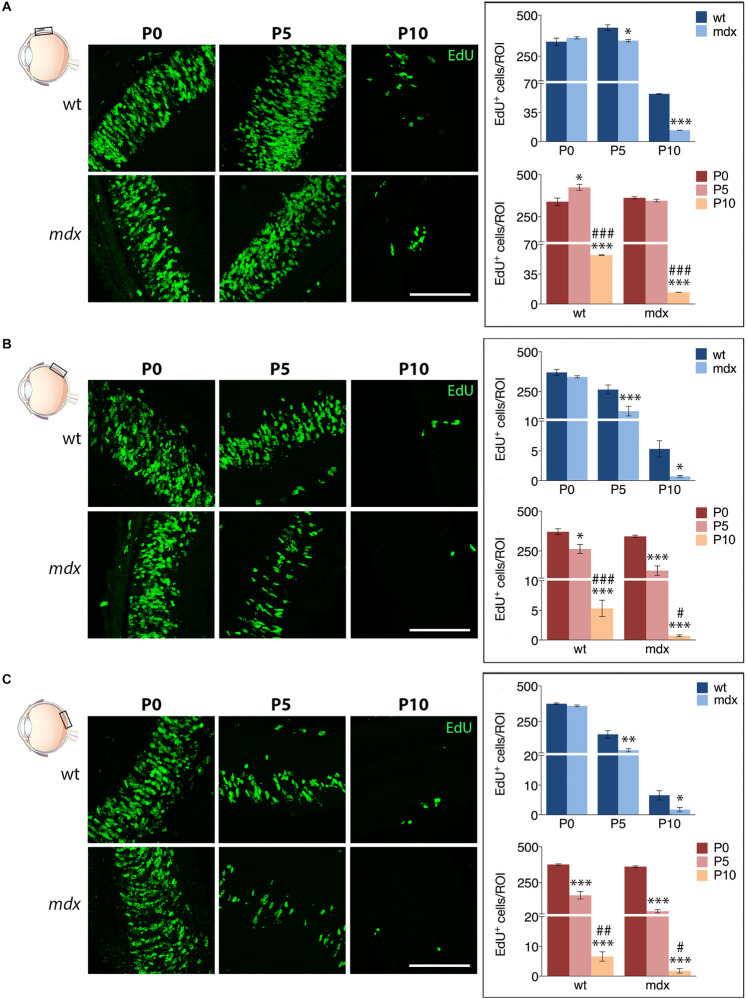
Lack of Dp427 reduces the number of EdU^+^ proliferating cells in P5 and P10 *mdx* mice compared to wild type. On the left side, representative images of EdU^+^ cell (green) distribution in the anterior **(A)**, central **(B)**, and posterior **(C)** regions of the retina of P0, P5, and P10 wild type (wt) and *mdx* mice; nuclei are stained in blue (Hoechst). At P0–P5, proliferating cells mostly occupy the neuroblastic cell layer. On the right side, comparative analyses of the number of EdU^+^ proliferating cells/region of interest (ROI) between wt and *mdx* age-matched mice (top graphs) and across post-natal days within the same genotype (bottom graphs). Data are analyzed by either two-way ANOVA test Sidak *post hoc* (top graphs) or one-way ANOVA test Tukey *post hoc* (bottom graphs) and represented as the mean ± SEM. ^∗^*p* ≤ 0.05, ^∗∗^*p* ≤ 0.01, ^∗∗∗^*p* ≤ 0.001 (*mdx* vs. wt; P5, P10 vs. P0); ^#^*p* ≤ 0.05, ^##^*p* ≤ 0.01, ^###^*p* ≤ 0.001 (P10 vs. P5). *n* = 3–6 independent experiments. Scale bar: 100 μm.

We then compared the number of EdU^+^ cells counted in each specific region between age matched wild type and *mdx* mice (Figure 10). The results showed that at P5 and P10, the number of proliferating cells in all three regions (A, M, and P) of the *mdx* mouse retina was significantly lower (*p* ≤ 0.05 – *p* ≤ 0.001) compared to wild type (Figures 10A–C, top graphs), as also evident from the corresponding representative photographic fields (Figures 10A–C, left). No differences were, instead, detected at P0 (Figures 10A–C, top graphs). Furthermore, all regions showed a stage-dependent decrease of EdU^+^ cells between P0 and P10 in both genotypes (Figures 10A–C, bottom graphs). Of note, we detected a limited, but significant increase (*p* ≤ 0.05) of EdU^+^ cells in the A region of the retina in P5 wild type mice compared to P0, which was not present in *mdx* mice (Figure 10A, bottom graph).

### The Number of Apoptotic Cells Is Reduced in P10 *mdx* Mouse Retina Compared to Wild Type

**FIGURE 11:**
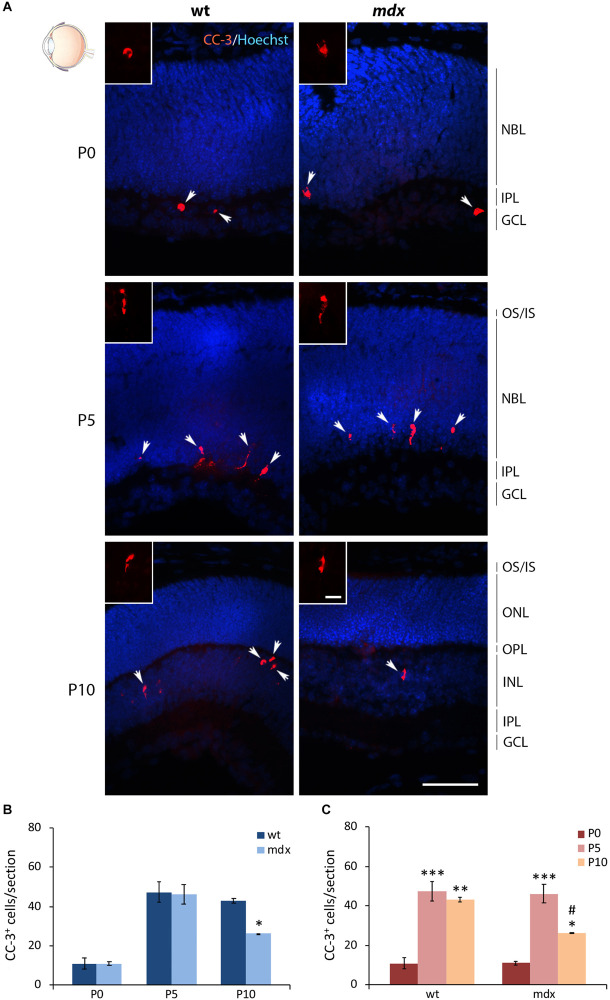
Lack of Dp427 determines a reduction in the number of cleaved caspase-3-immunopositive apoptotic cells during retinogenesis of P10 *mdx* mice compared to wild type. **(A)** Representative images of horizontal (N–T) retina cryosections of P0, P5, and P10 wild type (wt) and *mdx* mice immunolabeled for cleaved caspase-3 (CC-3). Nuclei are stained in blue (Hoechst). **(B,C)** Comparative quantitative analysis of the number of CC-3^+^ cells/section between wt and *mdx* age-matched mice **(B)** and across post-natal days within the same genotype **(C)**. Counts are made along the entire retinal perimeter. Data are analyzed by either two-way ANOVA test Sidak *post hoc* **(B)** or one-way ANOVA test Tukey *post hoc* **(C)** and represented as the mean ± SEM. ^∗^*p* ≤ 0.05, ^∗∗^*p* ≤ 0.01, ^∗∗∗^*p* ≤ 0.001 (*mdx* vs. wt; P5, P10 vs. P0); ^#^*p* ≤ 0.05 (P10 vs. P5). *n* = 3–5 independent experiments. Scale bar: 50 μm; insets: 10 μm. OS, outer segment of photoreceptors; IS, inner segment of photoreceptors; NBL, neuroblastic cell layer; ONL, outer nuclear layer; OPL, outer plexiform layer; INL, inner nuclear layer; IPL, inner plexiform layer; GCL, ganglion cell layer.

Another important physiological aspect to be taken into consideration is the occurrence of programmed apoptotic cell death that, in the mammalian retina, reaches the highest rate between P5 and P10 (Vecino et al., 2004). We, therefore, quantified the number of cells immunopositive for the cleaved caspase 3 (CC-3), a classic apoptotic cell marker, at P0, P5, and P10 (Figure 11A). In each section the count was made on the whole retina. The number of apoptotic cells between aged-matched wild type and *mdx* mice was not different at P0 and P5, while it was significantly lower in P10 dystrophic rodents compared to wild type (Figure 11B). Analysis of the number of apoptotic cells within each genotype showed an expected significant increase of dying cells between P0 and P5–P10 mice (Figure 11C). This, however, was followed by a significant decrease in the number of apoptotic events between P5 and P10 in *mdx*, but not in wild type mice, in agreement with a different number of apoptotic cells observed in P10 wild type and *mdx* mouse retinas (Figure 11C).

### The Gene Expression Levels of Dtnb (β-Dystrobrevin), Capn3 (calpain3), and Id3 (Inhibitor of DNA Binding 3) Are Significantly Reduced in E18, P0, and P5 mdx Mouse Retina Compared to Wild Type

**FIGURE 12:**
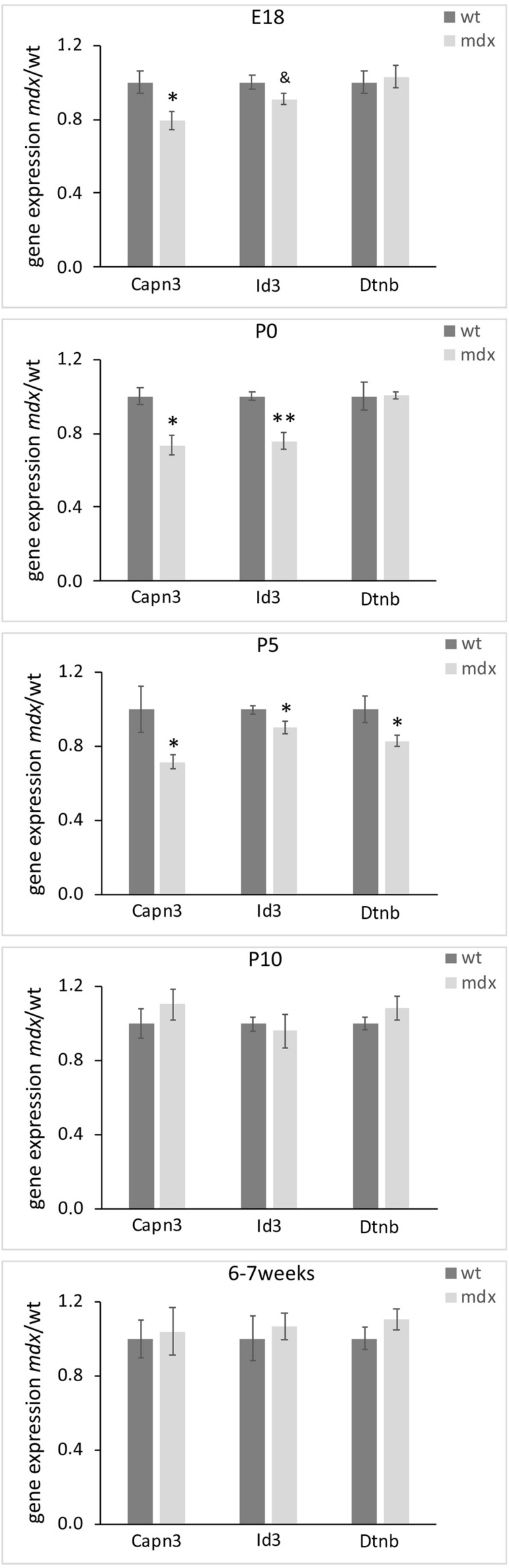
Lack of Dp-427 affects *Capn3*, *Id3*, and *Dntb* gene expression during pre- and post-natal retinogenesis. Retinal gene expression of *Capn3*, *Id3*, and *Dntb*, evaluated by real time RT-PCR, is significantly reduced in E18 (*Capn3*, *Id3*) and P0–P5 (all three genes) in *mdx* mice compared to wild type (wt). Data are analyzed by the two-tails Student’s *t*-test and represented as the mean ± SEM. ^&^*p* < 0.06; ^∗^*p* ≤ 0.05, ^∗∗^*p* ≤ 0.01 (*mdx* vs. wt). *n* = 3–6 independent experiments.

We finally sought to identify whether *mdx* mice could bear gene expression changes. To this aim, we employed real-time RT-PCR on dissected retinal tissues to compare, in wild type and *mdx* mice, the relative transcript levels of a cohort of genes involved in critical aspects of retinal development, such as cell specification, stress response, neuroprotection, apoptosis, cytoskeletal dynamics, and cell signaling (Supplementary Table S1). To identify transcriptional changes that may precede alternations in the postnatal retina, this analysis was also performed on E18 mice. Among the genes taken into consideration, we observed a significant downregulation of *Capn3* and *Id3* gene expression in E18, P0, and P5 *mdx* mouse retinas compared to wild type. At P5, we also detected a transient downregulation of *Dtnb*, which encodes for one of the proteins associated to the DGC (*p* ≤ 0.05) (Figure 12). No changes in the expression levels of these genes in wild type and *mdx* mouse retinas were found at later stages (P10 and 6–7 weeks) (Figure 12 and Supplementary Table S1).

## Discussion

An aspect of DMD pathophysiology is the manifestation, in both patients and animal models, of a variety of cognitive dysfunctions, behavioral and neurological disorders, neuron physiology abnormalities, and sensory defects (Mehler, 2000; Anderson et al., 2002; Chen et al., 2002; Cyrulnik and Hinton, 2008; Hinton et al., 2009; Waite et al., 2009; Ricotti et al., 2016b; Lombardi et al., 2017). Type, complexity and severity of the clinical picture relies not only on the lack of Dp427 but also on the lack of some or all its short isoforms (Doorenweerd et al., 2017). However, independently of whether one or more dystrophin isoforms underlie a specific phenotype, it is nowadays clear that nervous system disabilities in DMD originate from developmental dysfunctions, the progression of which is difficult to ascertain because of early death of patients (Doorenweerd et al., 2017).

### Late Retinogenesis in *mdx* Mice Is Characterized by Transient Alterations in the Progression of AC Maturation and VGluT1 Expression, Which May Concur to the Final Reduction in the Number of RGCs

Altered electroretinogram, reduced spatial luminance contrast and red-green color vision impairment have been described in both DMD patients and mice with DMD gene deletions downstream of exon 30 (lacking all dystrophin isoforms) (D’Souza et al., 1995; Costa et al., 2007; Costa et al., 2011; Barboni et al., 2013). These defects are absent or much reduced in adult *mdx* mice, the animal model used in this study, characterized by one single mutation in the exon 23 of the DMD gene and, therefore, lacking the sole Dp427 (Costa et al., 2007, 2011; Tsai et al., 2016; Bucher et al., 2019). For several years, our interest has been focused on the neurodevelopmental aspects of DMD, and specifically on the role that full length dystrophin plays in neuron physiology. In particular, we have previously reported, in *mdx* mice, reduced sensitivity of sympathetic neurons to nerve growth factor (Lombardi et al., 2008, 2017), a slowdown in axonal growth during development and regeneration after injury because of altered growth cone dynamics (Lombardi et al., 2017), increased neuronal loss in association with the natural occurring cell death (De Stefano et al., 2005), and reduction and/or alteration of peripheral sympathetic innervation (De Stefano et al., 2005; Lombardi et al., 2008). This brought us to investigate an aspect of vision physiology in DMD different from those so far analyzed, i.e., whether, despite the absence of gross visual defects in *mdx* mice, lack of Dp427 may alter, temporarily or permanently, specific aspects of late retinogenesis.

Expression of Dp427 has been demonstrated in the retina of human and several animal models (Wersinger et al., 2011), but to our knowledge only a few studies have analyzed its expression during development (E19 rats; E14 chick) (Rodius et al., 1997; Blank et al., 2002) and early post-natal days (P1 mice) (Bucher et al., 2019). We confirmed the expression of Dp427 at E18 by RT-PCR and at the early postnatal days considered in this work (P0, P5, and P10) by a Western immunoblot analysis (Supplementary Figure S1). In agreement with the available literature, in the wild type mouse retina, Dp427 mRNA was expressed since E18, and after birth its protein levels increased from P0 to P10; as expected, Dp427 was absent in *mdx* mouse retina homogenates (Supplementary Figure S1).

Retinogenesis is characterized by the migration of neuroepithelial progenitor cells, which go through a series of symmetric and asymmetric divisions, finely regulated in both space and time. In phase with cell cycle, progenitor cell migration follows different trajectories, depending on cell type commitment. A key aspect of this process is the interkinetic nuclear migration, according to which nuclei move in along the apico-basal axis (radial migration), dividing when they are close to the apical side and undergoing S phase in proximity to the basal one. Once post-mitotic, nuclei reach the final position in the appropriate retinal lamina and cells initiate differentiation. Details can be found in extensive reviews on retinogenesis (Baye and Link, 2008; Amini et al., 2017). Our first histological analysis showed no gross alterations, between wild type and *mdx* mice, concerning retina lamination, except for a temporary but significant difference in the thickness of the GCL at P0 and P5. The enlargement of the GCL at P0 in *mdx* mice compared to wild type may suggest that the differentiation process of this type of neurons, which is the first to be born during embryonic development (Heavner and Pevny, 2012; Amini et al., 2017), might be somehow altered in the dystrophic phenotype. Neuroblasts may prematurely stop earlier cell division, engulf in the basal side of the retina, and the development of their dendritic arborizations might be delayed. Supporting this hypothesis is the reversal of this phenotype at P5, when the thickness of the GCL in wild type mice increases, following the correct time schedule of cell differentiation. RGCs, however, are not the only cell type within this retinal layer, as ACs are also present and may contribute directly to these differences between the two genotypes. As a matter of fact, RGCs do not express Dp427, which instead has been described in both ACs, which are born concomitantly to RGCs, and BCs, which are born between P0 and P7 (Wersinger et al., 2011). By affecting the linkage of ECM proteins and actin cytoskeleton through the weakening of the DGC, in these types of retinal neurons the absence of Dp427 could interfere with the stabilization of membrane receptors for neurotransmitters (Knuesel et al., 1999; Zaccaria et al., 2000; Del Signore et al., 2002) and for other molecular cues (Lombardi et al., 2008, 2017), and/or alter appropriate cytoskeletal dynamics (Lombardi et al., 2017), as demonstrated for other central and autonomic neurons. These events may hamper one or more aspects related to AC and BC differentiation, among which structural and/or functional synaptic connectivity. As both cell types make contact on RGCs, this may in turn affect proper timing of RGC differentiation and/or survival, as discussed below.

A characterization of late retinogenesis through a progressive (from P0 to 6–7 weeks) quantitative analysis of the number of different retinal cell types is in line with these first observations. Overall, the time course of expression of all the cell markers used to identify specific cell types does not differ between wild type and *mdx* mice. In both genotypes, cell markers appear at the appropriate pre- or post-natal days, and the number of immunopositive cells either increases (commonly neuron maturing after birth concomitantly with eye enlargement) or decreases (neurons born before birth, when eyes are smaller), according to what is expected from literature data on visual map construction and refinement (Cayouette et al., 2006; Centanin and Wittbrodt, 2014; Nguyen-Ba-Charvet and Chédotal, 2014). This suggests that the time of commitment of the different RPCs into a specific cell type, a highly complex and diversified process (Cayouette et al., 2006; Goolsby et al., 2012; Centanin and Wittbrodt, 2014; Hoon et al., 2014; Amini et al., 2017), is not influenced by the absence of Dp427. Nonetheless, we observed a few intriguing differences between the two genotypes in the number of GABA^+^ ACs and CR^+^ RGCs. In particular, we report a significantly higher number of GABAergic ACs in P5 *mdx* mouse GCL compared to wild type, whereas this same parameter becomes significantly lower in P10 *mdx* mice. In these mutants, also the percentage of VGluT1 FA/ROI, a marker for ribbon synapse formation (Sherry et al., 2003), is lower compared to wild type. In the meanwhile, although no changes in the number of RGCs immunopositive to CR are revealed between the two genotypes at early postnatal days (when eyes are still closed), significantly less CR^+^ cells are present in the GCL of adult *mdx* mice compared to wild type. On the one hand, these results are in accord with all the studies identifying important roles for isoforms other than Dp427 in adult retina physiology, but on the other hand, they suggest that full length dystrophin may be more involved in modulating specific transitions during retinogenesis, which might induce changes too subtle to be revealed if not specifically addressed. Our hypothesis is that early and, possibly, temporary alterations to the connectivity between ACs-RGCs, ACs-BCs, and ACs-ACs, due to pre- and post-synaptic lack of Dp427, might affect some aspects of early and late retinogenesis.

As said before, photoreceptors, BCs and ACs of wild type mice express Dp427 at both pre- and post-synaptic sites (Wersinger et al., 2011). In particular, it has been demonstrated that the Dp427-DGC, along with specific ECM proteins (i.e., pikachurin), is required for the proper synaptic connections between photoreceptors and BCs (Sato et al., 2008; Omori et al., 2012). During retinogenesis, lack of Dp427 may cause a delay in the maturation and organization of ribbon synapses between photoreceptors and BCs. This delay, although not harming *per se*, might adversely affect other aspects of retinal connectivity. In particular, it is known that both pre- (E17–E21) and early post-natal (P0–P11) phases of retinogenesis of the most diverse animal models, are shaped by an intrinsic pattern of spontaneous and rhythmic bursting activity, which rapidly spreads across the retina in waves (Galli and Maffei, 1988; Maffei and Galli-Resta, 1990; Sernagor and Mehta, 2001; Firth et al., 2005; Xu et al., 2011). This wave activity is crucial for the proper Hebbian refinement of RGC visual connections (Goodman and Shatz, 1993; Wong, 1999; Sernagor and Mehta, 2001; Xu et al., 2011) and is mainly driven by two excitatory neurotransmitters: acetylcholine (ACh) and GABA, both released (or co-released) by startburst (displaced) ACs (Bansal et al., 2000; Feller, 2002; Sernagor et al., 2003; Firth et al., 2005; Leitch et al., 2005; Hennig et al., 2009). During the late stage of retinal waves (in mice, P11–P21), ACh is replaced by glutamate released by BCs (Firth et al., 2005), cholinergic neurotransmission switches from nicotinic receptors (nAChRs) to muscarinic receptors activity (Zhou and Zhao, 2000), and GABA activity shifts from excitatory to inhibitory (Sernagor et al., 2003; Leitch et al., 2005). The balance between excitatory and inhibitory inputs onto RGC dendrites operates the final refinements of visual paths (Soto et al., 2011). In the IPL, however, the network established by ACs is dense and complex, as one cell can establish synaptic contacts on the presynaptic terminals of BCs contacting RGCs (presynaptic inputs), on RGCs dendrites (postsynaptic input), and on other ACs (serial inputs) (Lee and Zhou, 2006; Franke and Baden, 2017; Jia et al., 2020). In particular, the crosstalk between ACs controlling distinct receptive fields provides a fine mechanism for feature-specific local (<150 μm) control of global (>1 mm) retinal activity (Jia et al., 2020). Wave activity is abolished by administration of nAChR antagonists (Feller, 2002) and is absent in mice knock out for the β2 subunit of nAChR (Bansal et al., 2000; McLaughlin et al., 2003). β2 knock out mice also lack refinement of the retinocollicular projections, which persists after the glutamate-dependent period of retinogenesis (Bansal et al., 2000; McLaughlin et al., 2003). We previously demonstrated that, in autonomic neurons of the superior cervical ganglion of *mdx* mice, lack of Dp427 affects clustering and membrane stabilization of post-synaptic nAChRs containing α3β2/ β4 subunits (Zaccaria et al., 2000; Del Signore et al., 2002) and determines a decrease in the fast intra-ganglionic transmission through this specific receptor sub-family (Di Angelantonio et al., 2011). In addition, a decrease in the response to a post-training nicotine challenge in a passive avoidance paradigm has been reported, reinforcing the idea of a role of the Dp427-DGC complex in the stabilization of certain sub-class of nAChRs (Coccurello et al., 2002). A similar role of Dp427 has also been demonstrated for GABA_A_ receptors in both cerebellum and hippocampus (Knuesel et al., 1999; Vaillend and Billard, 2002). Therefore, our results may suggest that, in *mdx* mice, dysfunction of β2-containing nAChR and/or GABA_A_ receptors during wave activity may induce some impairment in the cholinergic and GABAergic connectivity of ACs–ACs, ACs–BCs, and BCs–ACs couplings. This, in turn, could affect RGC plasticity, leading to a reduction in the number of CR^+^ mature neurons sometime between P10 and 6–7-week-old *mdx* mice. We cannot exclude, however, an effect also on the small population of displaced ACs, the 71% of which has been reported to be CR^+^ (May et al., 2008). An interesting aspect, which deserves to be investigated, is how this CR^+^ cell decrease reflects in RGC projections to higher visual centers, information which will help in settling the matter on whether this reduction is concrete and/or functionally invalidating.

### Lack of Dp427 May Be Responsible for the Observed Reduction in RPC Proliferation During Late Retinogenesis

Dp427 in muscle stem cells is an important regulator of cell polarity and asymmetric division, and its absence in DMD patients disrupts intracellular localization of cell polarity factors, with consequent reduction in asymmetric divisions, loss of polarity, impaired mitotic spindles, and prolonged cell divisions (Dumont et al., 2015). Similarly, the brain of DMD patients is characterized by several abnormalities consequent to altered neuronal migration, orientation, and polarity (Mehler, 2000). These may result from intrinsic alterations in neural progenitor cells, due to defective cytoskeletal dynamics and of its connectivity to ECM proteins, reduced stabilization of intracellular signaling pathways, and/or alteration in Ca^2+^ influx (Mehler, 2000). Studies on hippocampal adult neurogenesis in *mdx* mice have further evidenced prolonged cell proliferation and consequent suppression of neuronal differentiation (Deng et al., 2009). In this study we explored for the first time the role of Dp427 on RPC proliferation during late retinogenesis. Our results demonstrate that although the anterior (high) to posterior (low) gradient of proliferating cells (EdU^+^) along the entire retina is similar between the two genotypes, their number is always significantly lower in P5 and P10 *mdx* mice compared to wild type. To date, it is not known whether RPCs express Dp427 independently from the type of neuron they will generate and remains an interesting aspect to investigate. However, our results indicate that absence of Dp427 may affect the timing and/or the number of cell divisions during retinogenesis. Once again, this would possibly rely on the altered cytoskeleton-DGC-ECM coupling, which affects cytoskeletal dynamics, delocalizes intracellular factors important for proper instructive signaling, and ultimately may end in altered cell migration and/or polarization, or interkinetic nuclear translocation, terminating cell divisions earlier than in the wild type. We observed a similar deregulation also for the apoptotic process, which was significantly reduced in P10 *mdx* mice compared to wild type. In the retina, apoptosis has several key functions, among which elimination of neurons that fail to make functional synaptic contacts with their targets, and dismissal of transient cell populations playing an important function only during a particular developmental stage (Vecino et al., 2004; Vecino and Acera, 2015). Considering the significant and prolonged reduction in cell proliferation observed in dystrophic mice, a lower apoptotic rate in the final period of this peculiar neuronal pruning would probably represent a physiological consequence or, as discussed in the following section, the result of changes in the expression of genes involved in the apoptotic process.

**FIGURE 13:**
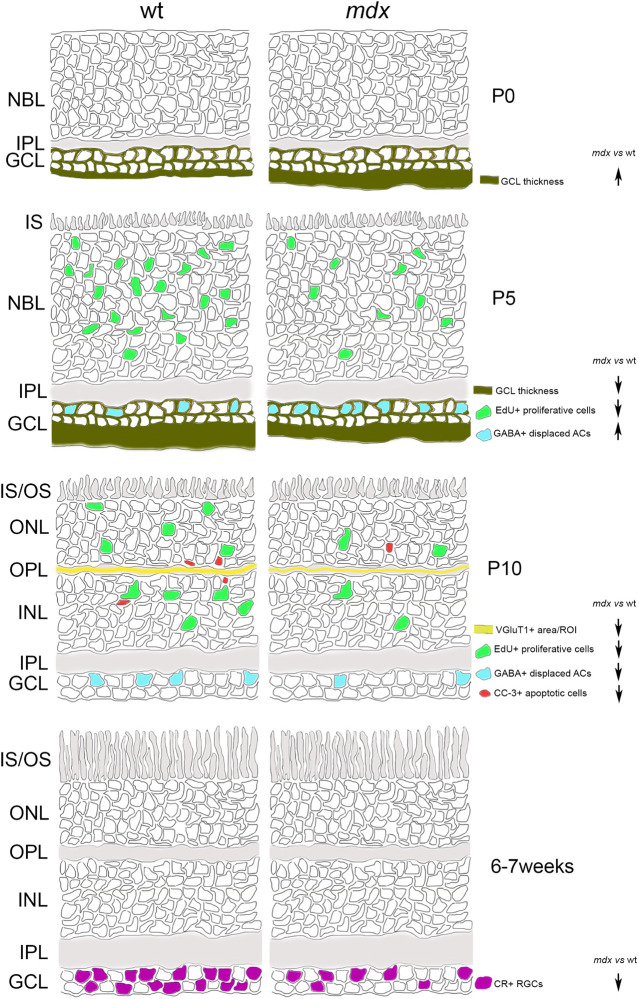
Schematic drawing depicting the main suggested alterations in *mdx* mouse retina during post-natal retinogenesis and adulthood. ACs, amacrine Cells; CR, calretinin; CC-3, cleaved caspase-3; GABA, gamma aminobutyric acid; GCL, Ganglion Cell Layer; INL, inner nuclear layer; IPL, inner plexiform layer; IS, inner segment of photoreceptors; NBL, neuroblastic cell layer; ONL, outer nuclear layer; OPL, outer plexiform layer; OS, outer segment of photoreceptors; RGCs, Retinal Ganglion Cells; VGluT1, Vesicular Glutamate Transporter 1; wt, wild type.

A graphical summary of the main results so far discussed is depicted in Figure 13.

### Lack of Dp427 Affects the Expression of Genes Related to Retinal Development

As previously demonstrated in autonomic neurons of the superior cervical ganglion (Licursi et al., 2012), lack of Dp427 determines direct and/or indirect modulation of selected transcriptional networks. The panel of genes investigated in the present study was focused on those encoding proteins important in ocular development and cell signaling, as well as proteins related to the DGC. Among them, three genes were temporarily downregulated in *mdx* mice compared to wild type during late retinogenesis: *Dtnb*, *Capn3*, and *Id3*, all coherent with the instructive role we suppose Dp427 plays during retinogenesis.

Dtnb is part of the DGC and, in the retina, is highly expressed by photoreceptors, where it concentrates in the presynaptic compartment of the ribbon synapses, according to the Dp427-DGC localization (Ueda et al., 2000). Therefore, the observed reduction in *Dtnb* gene expression may reflect a temporary altered pattern of DGC aggregation at synaptic level, which is also in agreement with the hypothesis of a delay in ribbon synapse maturation deduced by the VGluT1 immunofluorescence data. The absence of alteration in photoreceptor-HC transmission in adult *mdx* mice (Costa et al., 2007; Costa et al., 2011; Tsai et al., 2016; Bucher et al., 2019) would suggest that these alterations may be confined to the first stages of synapse formations, as compensated later on by the intervention of the other two dystrophin isoforms (Dp260, Dp140). Capn3 is a proteolytic enzyme activated during neuronal cell death (Nakajima et al., 2006) that interacts indirectly with the DGC. *Capn3* mutations have been found in the limb girdle muscular dystrophy (LGMD) type 2A (Kramerova et al., 2004). As for *Dtnb*, the reduction in *Capn3* expression observed in E18, P0, and P5 *mdx* mice is coherent with a destabilization of the DGC in late retinogenesis and with the observed reduction in apoptotic cells. Finally, Id3 is a member of the Id helix-loop-helix protein family that has been described as a positive regulator of cell proliferation during development (Norton et al., 1998; Kee and Bronner-Fraser, 2005). Id3 has been detected in RGCs and ACs of the post-natal and adult mouse retina, where it plays diversified roles, including the regulation of multipotent stem cells proliferation, and of terminal differentiation and maintenance of RGCs and ACs (Yeung and Yip, 2005). Therefore, the reduction in *Id3* gene expression observed in E18, P0, and P5 *mdx* mice is coherent with the reduction in cell proliferation observed at P5 and P10, as well as with the alterations in AC differentiation during late retinogenesis and the reduction in RGCs in adult animals.

## Conclusion

In conclusion, our data on the *mdx* mouse late retinogenesis show that lack of Dp427 alone does not induce major anatomical alterations, retinal cell disorganization, or extensive cell loss; this is in agreement with the literature data reporting on visual alterations in DMD patients and animal models. In fact, it is common opinion that among all the dystrophin isoforms expressed by retinal neurons, Dp427 is the most dispensable when analyzing processes as scotopic, contrast, and green-red color vision. However, by studying the pattern of lamination (histological analysis), cell proliferation (EdU labeling), apoptosis (CC-3 immunolabeling), neuronal differentiation (cell-specific marker immunolabeling) and gene expression during late retinogenesis (P0–11) and in adult mice, it appears clear that Dp427 is mainly, though not exclusively, involved in neurodevelopmental processes, as also reported for other central and autonomic neurons. Neural alterations during development, though, may be compensated during growth or underlie adult physiological dysfunctions, and this makes the difference on the role that Dp427 plays in different regions of the nervous systems. In this study, only a reduction in the number of CR^+^ RGCs is observed in adult *mdx* mice, while all other dissimilarities with respect to the wild type, seen during retinogenesis, appear to stabilize to normal levels. This leaves open the question, an object of future studies, of whether *mdx* mice present alterations in the ultrastructural organization of intraretinal synapses and/or in the topography of RGC projections to the superior colliculus.

## Data Availability Statement

Datasets are available on request: the raw data supporting the conclusions of this article will be made available by the authors, without undue reservation.

## Ethics Statement

The animal study was reviewed and approved by OPBA (Organismo Preposto al Benessere Animale) and by Ministero della Salute.

## Author Contributions

MD designed and directed the research. GL designed and directed the gene expression experiments. IP, FC, and NG performed the research. MD, IP, and GL wrote the manuscript. All authors contributed to the article and approved the submitted version.

## Conflict of Interest

The authors declare that the research was conducted in the absence of any commercial or financial relationships that could be construed as a potential conflict of interest.

## Footnotes

1 http://primer3.ut.ee
