# Supplementary material for: Dystrophin Is Required for the Proper Timing in Retinal Histogenesis: A Thorough Investigation on the mdx Mouse Model of Duchenne Muscular Dystrophy
Source: Front Neurosci. 2020 Aug 31;14:760. doi: 10.3389/fnins.2020.00760 (PMC7487415; doi:10.3389/fnins.2020.00760)
Supplement: Supplementary file 1 [file Table_1.docx]

Supplementary Table 1. List of genes analyzed by real time RT-PCR in E18, P0, P5 and P10 wild type and *mdx* mouse retina and the primers used. The wild type (w) and *mdx* (m) primers aiming at demonstrating the presence of transcripts with the wild-type and *mdx* dystrophin sequences, verified by RT-PCR, are also listed. On the right side of the table, the presence or absence of gene expression changes in *mdx* mice compared to wild type are indicated.

|  |  |  | **Gene expression in *mdx* mice compared to wild type** | | | | |
| --- | --- | --- | --- | --- | --- | --- | --- |
| **Gene** | **Gene Description** | **Primers** | **E18** | **P0** | **P5** | **P10** | **6-7 wks** |
| Capn3 | Mus musculus calpain 3 (Capn3), transcript variant b, mRNA | F: CTCTTGGTGCACAGCCTGAT  R: GCCATCTACAGGTGCTATGGA |  |  |  | = | = |
| Wnt3 | Mus musculus wingless-type MMTV integration site family, member 3 (Wnt3), mRNA | F: CGCTCAGCTATGAACAAGCA  R: CACCAGCAGGTCTTCACCTC | = | nt | = | nt | nt |
| Wnt5a | Mus musculus wingless-type MMTV integration site family, member 5A (Wnt5a), mRNA | F: AATCCACGCTAAGGGTTCCT  R: GAGCCAGACACTCCATGACA | = | nt | = | nt | nt |
| Wnt5b | Mus musculus wingless-type MMTV integration site family, member 5B (Wnt5b), mRNA | F: CCGAGAGCGTGAGAAGAACT  R: GGCGACATCAGCCATCTTAT | = | nt | = | nt | nt |
| Wnt7b | Mus musculus wingless-type MMTV integration site family, member 7B (Wnt7b), mRNA | F: ATGCCCGTGAGATCAAAAAG  R: GTGGTCCAGCAAGTTTTGGT | = | nt | = | nt | nt |
| Wnt13 | Mus musculus wingless-type MMTV integration site family, member 2B (Wnt2b), mRNA | F: CACCCGGACTGATCTTGTCT  R: GCCACAACACATGATTTCACA | = | nt | = | nt | nt |
| Atf4 | Mus musculus activating transcription factor 4 (Atf4), mRNA | F: CTGGCCAAGGAGATCCAGTA  R: CAAGCACAAAGCACCTGACT | = | nt | = | nt | nt |
| Areg | Mus musculus amphiregulin (Areg), mRNA | F: CATCGGCATCGTTATCACAG  R: ACAGTCCCGTTTTCTTGTCG | = | nt | = | nt | nt |
| Gsn | Mus musculus gelsolin (Gsn), mRNA | F: TTGGAAAAGACTCCCAGGAA  R: CCCTGCCTAACGACTGTGAT | = | nt | = | nt | nt |
| MKnK2 | Mus musculus MAP kinase-interacting serine/threonine kinase 2 (Mknk2), mRNA | F: ACCCTCCCAGTCCAAGCTG  R: GGCAGGGGCATATGTACAAG | = | nt | = | nt | nt |
| p75NTR | Mus musculus nerve growth factor receptor (TNFR superfamily, member 16) (Ngfr), mRNA | F: CATCTCTGTGGACAGCCAGA  R: CAGCTTCTCGACCTCCTCAC | = | nt | = | nt | nt |
| Id3 | Mus musculus inhibitor of DNA binding 3 (Id3), mRNA | F: AGAGGAGCTTTTGCCACTGA  R: GAGAGAGGGTCCCAGAGTCC |  |  |  | = | = |
| Lef1 | Mus musculus lymphoid enhancer binding factor 1 (Lef1), mRNA | F: CAGCTTTATCCAGGCTGGTC  R: GCTGTCATTCTGGGACCTGT | = | nt | = | nt | nt |
| Npy | Mus musculus neuropeptide Y (Npy), mRNA | F: AGAGATCCAGCCCTGAGACA  R: GATGAGGGTGGAAACTTGGA | = | nt | = | nt | nt |
| Pcp4 | Mus musculus Purkinje cell protein 4 (Pcp4), mRNA | F: CGACCAACGGAAAAGACAAG  R: CTGAGACTGAATGGCCACAG | = | nt | = | nt | nt |
| Vim | Mus musculus vimentin (Vim), mRNA | F: CCTGGAGTCACTTCCTCTGG  R: GGTCATCGTGATGCTGAGAA | = | nt | = | nt | nt |
| Axin2 | Mus musculus axin 2 (Axin2), mRNA | Axin2_1_SG QuantiTect Primer Assay QT00126539 (Qiagen, Hilden, Germany) | = | nt | = | nt | nt |
| Glut6 | Mus musculus solute carrier family 2 (facilitated glucose transporter), member 6 (Slc2a6), mRNA | F: TTTCGTCCCCATCTTCTTTG  R: GACGTAGGTTTTGGGCACAT | = | = | = | = | nt |
| Brn3b | Mus musculus POU domain, class 4, transcription factor 2 (Pou4f2), mRNA | F: ATCGCCGAAAAGCTGGATCT  R: AAAGAGGGCGAAGGGAAAGG | = | = | = | = | nt |
| Rbpms | Mus musculus POU domain, class 4, transcription factor 2 (Pou4f2), mRNA | F: ACCTCAGTTCATTGCCAGGG  R: GTGAAGCGGGGTAGGTGAAA | = | = | = | = | nt |
| Foxn4 | Mus musculus forkhead box N4 (Foxn4), mRNA | F: TATGCTGCCTACTCCACTGC  R: CCTTTCTTCCAGGCCGACAG | = | = | = | = | nt |
| Ptf1a | Mus musculus pancreas specific transcription factor, 1a (Ptf1a), mRNA | F: CAGCTAAAGTGTGGACCCCA  R: CAACCCGATGTGAGCTGTCT | = | = | = | = | nt |
| Crx | Mus musculus cone-rod homeobox (Crx), mRNA | F: TTTGCCAAGACCCAGTACCC  R: GCCCTACGATTCTTGAACCAGA | = | = | = | = | nt |
| Vsx2 | Mus musculus visual system homeobox 2 (Vsx2), mRNA | F: AAGCCAGAAGAAGAGGACGC  R: GGAGAAGAGCAGTTCCGAGG | = | = | = | = | nt |
| Otx2 | Mus musculus orthodenticle homeobox 2 (Otx2), mRNA | Otx2_2_SG QuantiTect Primer Assay QT01079771 (Qiagen, Hilden, Germany) | = | = | = | = | nt |
| Six3 | Mus musculus sine oculis-related homeobox 3 (Six3), mRNA | Six3_1_SG QuantiTect Primer Assay QT01045219 (Qiagen, Hilden, Germany) | = | = | = | = | nt |
| Pax6 | Mus musculus paired box 6 (Pax6), mRNA | Pax6_1_SG QuantiTect Primer Assay QT01052786 (Qiagen, Hilden, Germany) | = | = | = | = | nt |
| DG | Mus musculus dystroglycan 1 (Dag1), mRNA | F: CCTCCGTATGTTCCCCCTTA  R: TGTGCTAGGTCAGGTGTTGG | = | = | = | = | = |
| dystrobrevin | Mus musculus dyastrobrevin, beta (Dtnb), mRNA | F: TCCATCACCAACACCATGTC  R: AGCAGAAAGACGGACAGGAA | = | = |  | = | = |
| Pikachurin | Mus musculus EGF-like, fibronectin type III and laminin G domains (Egflam), mRNA | F: CATCATGGTGAACGGTTCCT  R: TTCTGGCTCCGTAGTCATCC | = | = | = | = | = |
| TrkA | Mus musculus neurotrophic tyrosine kinase, receptor, type 1 (Ntrk1), mRNA | F: GATGTGTGGAGCTTTGGGGT  R: CGTAGACATCAGGAGGGCAG | = | nt | = | nt | nt |
| TrkB | Mus musculus neurotrophic tyrosine kinase, receptor, type 2 (Ntrk2), mRNA | F: TGCTGGTGAAAATTGGGGACT  R: TCTCGGTGGTGAATTTCCTGT | = | nt | = | nt | nt |
| Eef1a1 | Eukaryotic Translation Elongation Factor 1 Alpha 1 | F: AGCTGGCAAAGTCACCAAGT  R: CCGTTCTTCCACCACTGA TT |  |  |  |  |  |
| *Dystrophin* | C57-specific primer (w) | F: GACACTTTACCACCAATGCG  R: TCAGATAGTTGAAGCCATTTTG |  |  |  |  |  |
| *Dystrophin* | *mdx*- specific primer (*m*) | F: GACACTTTACCACCAATGCG  R:CTCAGATAGTTGAAGCCATTTTA |  |  |  |  |  |

nt: not tested; =: unchanged expression; : decreased expression
