# Supplementary figures and images for: Dystrophin Is Required for the Proper Timing in Retinal Histogenesis: A Thorough Investigation on the mdx Mouse Model of Duchenne Muscular Dystrophy
Source: Front Neurosci. 2020 Aug 31;14:760. doi: 10.3389/fnins.2020.00760 (PMC7487415; doi:10.3389/fnins.2020.00760)

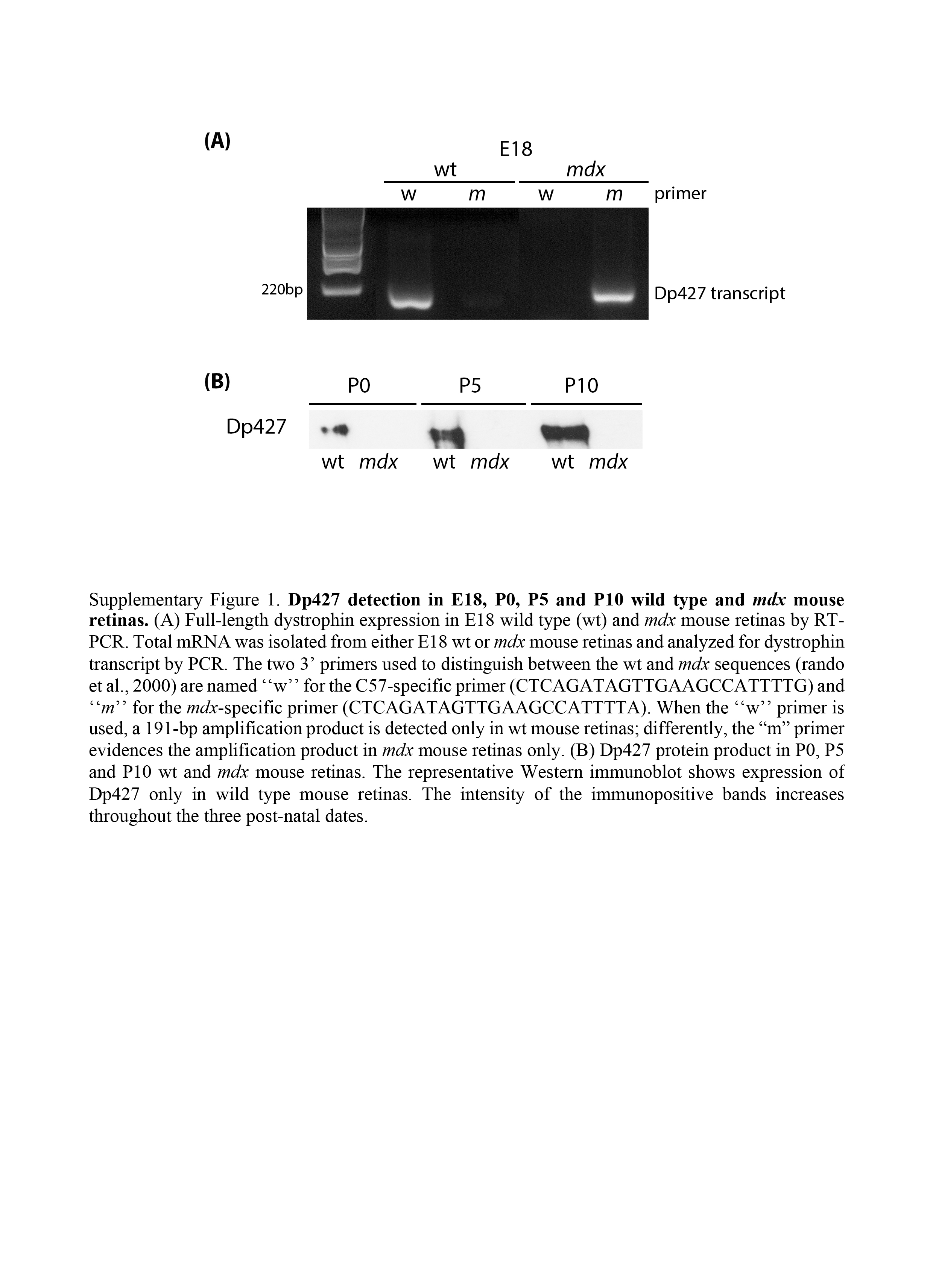

Supplement: Supplementary file 2 [file Image_1.JPEG]
